# Supplementary material for: Effective connectivity predicts distributed neural coding of perceptual decision confidence, uncertainty, and speed
Source: Imaging Neurosci (Camb). 2025 Jan 21;3:imag_a_00441. doi: 10.1162/imag_a_00441 (PMC12319975; doi:10.1162/imag_a_00441)
Supplement: Supplementary Material [file imag_a_00441-supp.pdf]

Supplementary Materials for

Effective connectivity predicts distributed neural coding of perceptual decision confidence, uncertainty and speed

Abdoreza Asadpour* and KongFatt Wong-Lin*

*Corresponding authors. Abdoreza Asadpour (a.asadpour@sussex.ac.uk); KongFatt Wong-Lin (k.wong-lin@ulster.ac.uk)

This file includes:

Supplementary Note 1
Table S1
Supplementary Note 2
Figs. S1 to S19
References

Supplementary Note 1

In this Supplementary Information, we provide detailed information and analyses that support the findings reported in the main manuscript. The supplementary materials encompass comprehensive description of our methods as well as tables and figures that collectively provide further details on the relationship between decision confidence levels, choice-based reaction times (RTs), and their neural correlates in perceptual decision-making. Below is a summary of each item.

Table S1 lists the fMRI, EEG and EEG-informed fMRI active brain regions that correlate with participants' decision confidence reports during the decision phase based on findings from previous studies, presenting, to date, a comprehensive overview of neural correlates associated with confidence in decision-making tasks. In particular, regions such as the ventral striatum, anterior cingulate cortex (ACC), supplementary motor area (SMA), and multiple frontal and parietal sites have been consistently implicated in decision confidence during stimulus presentation/decision formation phase.

Supplementary Note 2 covers the comprehensive methodological details which we presented succinctly in the paper.

Figures S1 to S19 show the results of our analysis of neural activity response under various conditions:

- **Fig. S1**: Statistical parametric map of the T-statistic (SPM{T}) maps show no significant differences in brain activation between low and high confidence sessions during the stimulation/response phase.
- **Fig. S2**: Significantly higher brain activation differences for high-vs-low confidence rating condition.

- **Fig. S3**: No significant differential brain activation during stimulation/response phase in high-vs-low confidence rating condition when excluding subjects with non-significant RT-confidence relationship.
- **Fig. S4**: No significant differential brain activation during stimulation/response phase in low-vs-high confidence rating condition when excluding subjects with non-significant RT-confidence relationship.
- **Fig. S5**: Significant sensory and movement related active regions against implicit baseline during stimulation phase.
- **Fig. S6**: No significant differential brain activation between correct and incorrect responses during stimulation/response phase. Error trials too low for statistical significance using family-wise error (FWE) correction.
- **Fig. S7**: No significant differential brain activation when comparing incorrect to correct choices during stimulation/response phase. Error trials too low for statistical significance using family-wise error (FWE) correction.
- **Fig. S8**: No significant differential brain activity for fMRI SPM{T} analysis for leftward-vs-rightward stimulus condition during stimulation/response phase.
- **Fig. S9**: No significant differential brain activity for fMRI SPM{T} analysis for rightward-vs-leftward stimulus condition during stimulation/response phase.
- **Fig. S10**: Scalp topography depicting differential EEG activity for high-vs-low confidence rating condition.
- **Fig. S11**: Predominant encoding of confidence rating by DP neural population in left precuneus, while encoding subjective uncertainty by SS neural population in

left superior frontal gyrus, illustrating the simultaneous neural encoding of decision confidence and uncertainty.

- **Fig. S12**: Higher brain activation associated with fast-vs-slow choice-based RT condition.
- **Fig. S13**: Significantly higher brain activity in specific regions for slow-vs-fast choice-based RT condition.
- **Fig. S14**: Scalp maps representing observed and model-predicted EEG activity during the stimulation/response phase for trials categorized by choice-based RTs.
- **Fig. S15**: Involvement of left precuneus in encoding response speed, suggesting its potential role in the objective evaluation of decision confidence.
- **Fig. S16**: Correlation of estimated source activity with confidence ratings and choice-based RTs in high confidence rating trials.
- **Fig. S17**: Estimated activities of inhibitory Interneurons (II) in left superior frontal gyrus associated with the encoding of subjective decision uncertainty in low confidence rating trials.
- **Fig. S18**: Estimated SP and DP activities in right superior parietal lobule correlate with subjective decision uncertainty.
- **Fig. S19**: In slow RT trials, left precuneus correlates with confidence rating, while estimated DP activities in right superior parietal lobule correlates with faster choice-based RTs.

Table S1. Brain region activations correlating with human participants' decision confidence during stimulation/decision formation phase and post-decision phases. Orange (black): positive (negative) correlation. Abbreviations of brain regions: angular gyrus (AG), inferior parietal gyrus (IPG), calcarine gyrus (CalG), rectal gyrus (RG), ventral striatum (VS), frontal eye field (FEF), medial prefrontal cortex (mPFC), precuneus (PreCUN), medial parietal (mPar), temporal pole (TP), striatum (Str), lateral orbitofrontal cortex (lOFC), anterior prefrontal cortex (aPFC), dorsomedial prefrontal cortex (dmPFC), inferior frontal gyrus (IFG), middle frontal gyrus (MFG), superior frontal gyrus (SFG), dorsolateral prefrontal cortex (dlPFC), ventromedial prefrontal cortex (vmPFC), supplementary eye field (SEF), inferior parietal lobule (IPL), intraparietal sulcus (IPS), middle occipital gyrus (MOG), medial temporal lobe (MTL), superior frontal cortex (SFC), supramarginal gyrus (SMG), anterior insula (aINS), parietal (Par), posterior (Post), cerebellum (Cer), thalamus (Thal), cuneus (Cun), lingual gyrus (Lin), fusiform gyrus (Fus), putamen (Put), medial frontal cortex (MFC), precentral gyrus (PreCG), postcentral gyrus (PostCG), paracentral lobule (ParCL), superior parietal lobule (SPL), basal forebrain (BF), hippocampus (HC), inferior frontal junction (IFJ), inferior temporal gyrus (ITG), superior temporal gyrus (STG), middle temporal gyrus (MTG), superior occipital gyrus (SOG), posterior cingulate cortex (PCC), rostro-lateral prefrontal cortex (rlPFC).

Publications	Task paradigm	Imaging modality	Task phase	Brain regions correlated with confidence
**Heereman et al. (Heereman et al., 2015)**	RDM	fMRI	Stimulation / decision formation	L AG, L IPG, CalG, R Lin, R Fus, vmPFC, RG, L posterior cingulate, SMA/dmPFC, R SFG, L Lin, L Fus, R PreCUN, SPL, R IPL, L IFG
**Hebart et al. (Hebart et al., 2016)**		fMRI	Stimulation / decision formation	VS, ACC, SMA, FEF, IFG, SPL, rMT+, R aINS, R PreCG
**Gherman and Philiastides (Gherman & Philiastides, 2018)**		EEG-informed fMRI	Stimulation / decision formation	Str, lOFC, ACC , Lateral occipital cortex (inferior), MFG (anterior), Occipital pole, R Cer, R ITG, SFG (SMA), dmPFC, R IFG, L PreCG

Publications	Task paradigm	Imaging modality	Task phase	Brain regions correlated with confidence
**Qiu et al. (Qiu et al., 2018)**		fMRI	Initial decision	No activation compared to control trials
**Li and Yang (Li & Yang, 2012)**	Glass pattern	fMRI	Stimulation / decision formation	R Post MFC, IPS, L SFG, L aINSr, L IFG, L IPL, L Post fusiform
**Gherman and Philiastides (Gherman & Philiastides, 2015)**	Face + Object	EEG	Stimulation / decision formation	dmPFC, Par cortex
**Qiu et al. (Qiu et al., 2018)**	Sudoku	fMRI	Initial decision	IFJ
**Jaeger et al. (Jaeger et al., 2020)**	Gap Location	fMRI	Stimulation / decision formation	BF, R SEFs, IPL, SMG, MFG, Cer, MOG, Put, visual Thal, and right PostCG
**Shapiro and Grafton (Shapiro & Grafton, 2020)**	Approach–avoidance task	fMRI	Stimulation / decision formation	STG, MTG, L Cun, SOG, R Lin, R SMG, R PostCG, R Inferior Par, R SMG, R Inferior Par, R MFG, R Median cingulate and paracingulate

Publications	Task paradigm	Imaging modality	Task phase	Brain regions correlated with confidence
				gyri, L Anterior cingulate and paracingulate gyri, R SFG, dorsolateral, R MFG, R PreCUN, R MFG
**Hoven et al. (Hoven et al., 2022)**	Gabor patches	fMRI	Stimulation / decision formation	vmPFC, PCC, dlPFC, rlPFC, aINS, R Put, R IFG, SMA, mid-ACC, ACC, IPL
**Post-decision brain activations**				
**Pereira et al. (Pereira et al., 2020)**	Box with dots	EEG-informed fMRI	Delay period between post-decision formation and confidence rating	Occipital, VS, L Put, L vmPFC, R HC, SMA, dACC, L SFC, L MFC, IFG, IPL, L aPFC, L MTL , L PreCUN
**Bang and Fleming (Bang & Fleming, 2018)**	RDM	fMRI	Unspecified	perigenual ACC
**Fleming et al. (Fleming et al., 2018)**		fMRI	Post-decision confidence rating	medial aPFC, PreCUN/ mPar, Temporal lobe, White matter, L Inf. Par, R Sup. Occipital,

Publications	Task paradigm	Imaging modality	Task phase	Brain regions correlated with confidence
				MFG, R PreCG, ParCL, R Cer, Occipital / inf. Par, Thal, pMFC, lateral aPFC
**Hilgenstock et al. (Hilgenstock et al., 2014)**	Grating Orientation Task	fMRI	Delay period between post-decision formation and confidence rating	superior medial gyrus bilateral, R aPFC, R dlPFC
**Morales et al. (Morales et al., 2018)**	Word-Shape recognition task	fMRI	Confidence/follow rating	left PreCG, left PostCG, Post midline VS, vmPFC, dACC/pre-SMA, Par cortex, bilateral PFC

Supplementary Note 2

Data description

An open concurrent EEG-fMRI dataset on perceptual decision confidence was utilised to investigate the active brain regions and effective connectivity of fMRI-informed EEG data (Gherman & Philiastides, 2020). The dataset comprises 24 participants aged 20–32 years; however, due to inconsistencies in the structural and functional data of the last participant, analyses were conducted on the remaining 23 participants. These participants were right-handed, had normal vision, and no history of neurological disorders (Gherman & Philiastides, 2018).

Participants discriminated the direction of coherent motion in RDM and rated their confidence. The RDM stimuli comprised white dots moving in a black background within a circular aperture, with a subset moving coherently to form the signal, and the rest moving randomly as noise. Task difficulty was controlled by adjusting the proportion of coherently moving dots, with the aim of maintaining overall performance at approximately 75% correct responses, individually calibrated for each participant during a separate training session using a 3-down-1-up staircase procedure. Each trial began with an RDM stimulus presented for a maximum of 1.2 seconds, during which participants made a left or right-directional discrimination using a button press with their right index finger. This was followed by a blank screen and a random delay of 1.5 to 4 s. Subsequently, participants rated their confidence on a white horizontal bar scale for 3 s. Trials ended with another random delay of 1.5 to 4 seconds. Each participant performed two experimental blocks of 160 trials each, corresponding to two separate fMRI runs. All

behavioural responses were executed using the right hand on an MR-compatible button box. Fig. 2A shows the sequence within a trial.

A 3-T Siemens MRI scanner was used to record two main task runs, each with 794 brain volumes. Functional data were acquired using a T2*-weighted gradient echo, echo-planar imaging (EPI) sequence with the following parameters: 32 interleaved slices, 0.3 mm gap, 3 × 3 × 3 mm voxel size, 70 × 70 matrix size, 210 mm field of view (FOV), 30 ms echo time (TE), 2000 ms repetition time (TR), and 80° flip angle. Additionally, a high spatial resolution anatomical volume was obtained at the end of the session using a T1-weighted sequence with these parameters: 192 slices, 0.5 mm gap, 1 × 1 × 1 mm voxel size, 256 × 256 matrix size, 256 mm FOV, 2300 ms TE, 2.96 ms TR, and 9° flip angle.

Simultaneously with the fMRI data, EEG data were collected using a 64-channel MR-compatible system from Brain Products, Germany, with the Brain Vision Recorder software at a 5000 Hz sampling rate. Electrodes were positioned according to the 10 – 20 system, with additional nasion, reference, and ground electrodes. In-line resistors ensured participant safety and input impedance was maintained below 25 kΩ. Data acquisition was synchronized with the MRI scanner, and experimental events and participant responses were also recorded and synchronized with the EEG data. For further details, please refer to the original study (Gherman & Philiastides, 2018).

Data preprocessing

The fMRI data preprocessing procedure applied to the open dataset includes slice-timing correction to adjust for differences in image acquisition times, high-pass filtering with a cutoff of 100 s to remove low-frequency noise, spatial smoothing using a Gaussian kernel of 8 mm to improve signal-to-noise ratio, and head motion correction to account

for participants' movements during the scan. Additionally, we used SPM12 (Penny et al., 2011) for realignment of the fMRI images, extraction of motion parameters, co-registration of the mean fMRI volumes with the structural data for each participant, and normalisation to standard brain space.

In the open dataset, EEG data had been preprocessed using MATLAB. Gradient artefacts were corrected by subtracting average artefact templates from the EEG signal, followed by a 12 ms median filter to remove residual spike artefacts. Standard EEG artefacts were corrected, and a 0.5 – 40 Hz band-pass filter was applied to remove DC drifts and high-frequency noise, with data then downsampled to 1000 Hz. Eye movement and cardiac-related artefacts were minimized using principal component analysis, with data baseline corrected by removing the average signal during the 100 ms prestimulus interval (Gherman & Philiastides, 2018). No additional preprocessing steps were applied in our study.

Data Analysis

In our study, the data analysis was systematically divided into two pivotal subsections, each crucial for our subsequent DCM analysis. The first subsection, BOLD-fMRI data analysis, was essential in identifying active brain regions and their specific roles in the decision-making process. The second subsection, EEG data analysis, served as a preparatory phase, wherein the EEG data were processed for the DCM analysis. Together, these analyses form the foundation for our DCM analysis.

BOLD-fMRI data analysis

To statistically analyse the functional data and extract active brain regions under different conditions, we utilised the generalised linear model (GLM) technique in the

SPM12 fMRI toolbox (Penny et al., 2011). Specifically, if Y is the BOLD response over time for one voxel, the GLM can be formulated as:

$$Y = X\beta + \varepsilon = X_1\beta_1 + X_2\beta_2 + \cdots + X_N\beta_N + \varepsilon \qquad (1)$$

where β is model parameters, X is the design matrix for N regressors inclusive of effects of interest and no interest, and ε is the residual error vector. The optimal β weights that minimise the squared error values are calculable as (De Martino et al., 2015):

$$\beta = (X'X)^{-1}X'Y \qquad (2)$$

SPM generates the fMRI time series by convolving a time series of delta functions representing the event onsets with HRF, its time derivatives, and factors determining the width of the HRF as basis functions (Penny et al., 2011). Standard HRF utilised extensively in SPM is a mixture of two or more gamma functions (Penny et al., 2011).

For functional data analysis, we employed first-level and second-level statistical analyses to delineate active brain regions under varying conditions. The first-level analysis was conducted within-participant calculations using T-contrast calculations (Penny et al., 2011), a method designed for fMRI signal analysis across multiple conditions, assuming normally distributed data. This step involved calculating the t-statistic for each brain voxel to discern differences between the means of two conditions (high vs. low confidence ratings and fast vs slow RTs). Following this, we adjusted the p-values utilising Bonferroni correction (Bonferroni, 1936) and applied a family-wise error (FWE) correction at a level of 0.05 (Penny et al., 2011). For the second-level analysis, data from all participants were pooled together to facilitate group-level T-contrast analysis.

We conducted GLM analyses across all experimental phases, including stimulation presentation, delay, and confidence rating, to extract active brain regions and compare

different conditions within and across these phases (Penny et al., 2011). BOLD signals, inclusive of three condition-specific regressors and six nuisance regressors for motion artefacts, were analysed at three levels: runs, participants, and groups.

During and across all phases, we calculated T-contrasts to compare epochs of high vs. low confidence ratings and fast vs. slow RTs. This identified regions with significant activity differences between these states (Penny et al., 2011). Confidence levels were categorised into high (≥7), medium (5, 6), and low (≤4). RTs were clustered into four groups using k-means clustering with IBM SPSS Statistics (Version 27) (IBM Corp., 2020), computed separately for each participant. This approach allowed us to capture the natural variability in RT distributions without imposing equal trial numbers across bins. Quartile binning was avoided as it would have constrained the groupings into equal-sized bins, potentially misrepresenting the natural variability in RTs. RTs below the second cluster centre were classified as fast, and those above the third cluster centre as slow. We employed T-contrasts to discern active brain regions during each phase, applying family-wise error correction ($p < 0.05$) to all calculations (Penny et al., 2011). The identified active brain regions will subsequently inform our DCM analysis. Given our interest in early neural correlates of decision confidence, we conducted a further analysis of the stimulation presentation phase.

EEG data analysis

Using the EEGLAB toolbox (Delorme & Makeig, 2004), we added the events to the dataset. Then we exported it to the SPM12 toolbox for further analysis. Within SPM12, we divided the EEG data into epochs (100 ms before the stimulus onset to 2s after the stimulus onset for each participant based on the onsets of the stimulation phase, and we

selected epochs corresponding to various conditions (high and low confidence ratings, fast and slow RTs) during the stimulation phase, and prepared them for DCM implementation. The dynamics from the EEG data will then be used to identify effective connectivity at the source level via DCM, guided by BOLD-fMRI data. Importantly, DCM creates a forward model to map estimated neural activity at the source level to scalp EEG activity using the leadfield matrix, rather than performing source localisation (David et al., 2006; Penny et al., 2011).

DCM analysis

In our study, we utilised DCM (Penny et al., 2011) to investigate the neural mechanisms underlying early confidence evaluation during perceptual decision-making. DCM enables us to explore directed connectivity between brain regions, thereby providing valuable insights into how these regions interact and contribute to confidence evaluation. To achieve a comprehensive analysis, we employed two types of DCM analyses: averaged ERP-DCM and trial-by-trial ERP-DCM. Averaged ERP-DCM analysis captures general trends and predominant patterns of neural connectivity across all trials, offering a robust overview of the brain's overall response during the task (David et al., 2006). Furthermore, we implemented an innovative trial-by-trial ERP-DCM approach, delving deeper into the individual trial dynamics. This allows us to dissect the variability and specifics of neural interactions in relation to early confidence evaluation. By integrating these two approaches, we aim to provide a holistic and nuanced understanding of the neural basis of early confidence evaluation in perceptual decision-making.

Averaged ERP-DCM analysis

In our study, the DCM analysis was instrumental in deciphering the neural mechanisms underlying perceptual confidence and decision-making processes. Our analysis began by meticulously defining the model space, a foundational step that delineates potential connectivity patterns among brain regions based on GLM results (Penny et al., 2011). Insights from the GLM analysis facilitated the formulation of hypotheses regarding possible forward and backward extrinsic connections between active cortical regions.

We were interested in modelling gamma band activities as they have been suggested to be related to perceptual decision-making (Donner et al., 2009). Thus, we selected the CMC neural model, which comprised four distinct neural populations per cortical column, namely SS neural population, II, DP, and SP neural populations (Bastos et al., 2012; Pinotsis et al., 2013). The CMC model is particularly suitable for modelling higher brain frequencies, including gamma activity, due to its inclusion of multiple neural populations within the cortical column, as opposed to conventional ERP-DCMs which may not adequately capture these higher frequencies (Pinotsis et al., 2013). These populations are intricately interconnected, as depicted in Fig. 2B, with each population playing a unique role in modulating neural activity.

For each participant and condition, we averaged the epochs corresponding to high and low confidence ratings, as well as fast and slow choice-based RTs, from the stimulation phase, which spanned from 50 ms prior to stimulus onset to 1.2 seconds after onset. However, our DCMs estimated neural activity until 800 ms post-stimulus onset to particularly investigate early neural correlates.

Neural activity is fundamentally influenced by the fluctuations in voltages and currents across different neural populations. In the CMC model, SP and DP neural populations predominantly affect post-synaptic potentials, serving as vital indicators of neural activity. Excitatory SS neural population contribute to feedforward input, while inhibitory interneurons modulate these dynamics by regulating the currents. A simplified illustration of these relationships is provided here (Fig. 2B), with a comprehensive mathematical depiction available in Pinotsis et al. (Pinotsis et al., 2013).

To probe the neural basis of early confidence evaluation, we employed a cumulative Gaussian signal as our stimulus input, similar to the approach used in FitzGerald et al. (FitzGerald et al., 2015), after also examining a Gaussian bump and sums of two logistic functions. However, the other functions could not estimate the EEG data optimally. The onset of the stimulus was set at approximately 200 ms post-stimulus (Fig. 2C), accounting for the delay in sensory information reaching cortical sensory regions, such as the parietal areas (Lamme & Roelfsema, 2000). Using SPM12, we linearly mapped the post-synaptic voltage, primarily from SP neural population, to scalp EEG activities through a cortical surface patch (Penny et al., 2011). By assigning Gaussian distributions to the parameters and varying extrinsic forward and backward connections (i.e., A^F and A^B) across different DCM models, we estimated the probability density of model parameters, utilising variational free energy and the Laplace approximation method (Penny et al., 2011).

Statistical analysis of averaged ERP-DCM

After calculating the inverse models in the model space, a BMS identified the winning model using the RFX procedure (Penny et al., 2011). BMS is a statistical method used to compare different models based on their likelihood given the observed data. It is an

essential step in model comparison as it informs us about the most probable model that explains the observed data. The RFX approach was chosen because it accounts for the variability between participants, assuming different performance mechanisms for different participants (Penny et al., 2011).

Considering each model's estimated posterior distribution given the observed data, one can utilise the BMA strategy to calculate the average over all models by averaging the posterior distributions of models weighted by their posterior model probability (Trujillo-Barreto et al., 2004). BMA is a valuable tool as it helps in obtaining a more accurate estimate by considering the uncertainty in model selection.

To investigate variability between participants, we employed PEB on participant-specific effective connectivity parameters retrieved from the winning DCM (Penny et al., 2011). PEB is a hierarchical Bayesian approach that provides participant-level estimated connection strengths and their uncertainty at the group level.

Trial-by-trial ERP-DCM analysis

In our study, following the averaged ERP-DCM analysis and identification of the winning model for each condition, we employed the trial-by-trial ERP-DCM approach to analyse and compare the neural dynamics of conditions (high vs low confidence rating and fast vs slow RT) during the stimulation phase. We used the connections from the winning model to train the trial-by-trial DCMs with the CMC model.

For each trial, we used the data from the whole epoch, which ranged from -50 ms to 1.2 s from the stimulation onset, to train the DCMs. This range was chosen to ensure that we captured all relevant neural activity during the stimulation phase. The initial parameters of the trial-by-trial DCMs were kept the same as those used in the averaged

ERP-DCMs. By employing this innovative approach, we aimed to gain a more comprehensive understanding of the neural basis of early confidence evaluation.

Statistical analysis of trial-by-trial ERP-DCMs

The statistical analysis of trial-by-trial ERP-DCM was performed to uncover shared neural mechanisms underlying behavioural responses. In our analysis, we were particularly interested in identifying time points in the activity of the four different neural populations in the CMC model—SS neural population, II, DP, and SP neural populations—within the extracted active brain regions (sources) for each condition that have a significant correlation with the behavioural data. We hypothesised that this relationship might exist, as indicated by previous research (Kepecs et al., 2008; Kiani & Shadlen, 2009).

SVR with linear and non-linear (Gaussian) kernels was utilised to establish associations between neural activity patterns and behavioural metrics (Drucker et al., 1996). After selecting the proper window length based on the three lowest RTs among the participants, we trained the SVR models for each participant for each condition, using all of the estimated neural population activities for trials corresponding to each condition.

The SVR models were then evaluated for their predictive performance. We then searched for the top 200 important features (time points) within this selected window. These features were then assessed for their importance in predicting behavioural outcomes using permutation importance (Breiman, 2001). This technique involved permuting each feature within the dataset while maintaining the total number of 1250 permutations. We also performed a sensitivity analysis on the form of perturbation to

determine if the relationship between the neural population activity and behavioural data (RT or confidence rating) is positive or negative (Hinch, 1991).

The significance of the predictive performances of the SVR models was then scrutinised using bootstrapping (Efron, 1979). This entailed resampling with replacement to construct distributions of predictive performances for each source and neural population. The significance of these performances was determined by calculating confidence intervals through bootstrapping 10,000 times, with a predefined threshold of 70% applied to evaluate the results. The 70% threshold is considered reasonable as it is above the 50% baseline, which is equivalent to random guessing in binary classification (James et al., 2013). Moreover, achieving a performance of 90% or higher might be unrealistic due to the complexity of neural data and substantial noise (Hastie et al., 2009).

After evaluating the performance of SVR models, we conducted sensitivity analysis to determine whether the relationship between neural population activity and behavioural data (RT or confidence rating) is positive or negative. This involved perturbing the data by adding a perturbation factor to the neural activity and re-evaluating the SVR models to observe any changes in the estimated subjective confidence rating or choice-based RTs.

Lastly, we identified significantly common features across subjects and conditions using a permutation test with 10,000 permutations (Fisher, 1935). The steps included performing a histogram count of all subjects to extract the data point repeats among subjects, undertaking 10,000 permutations, calculating a threshold for the counts with an alpha level of 0.05, and identifying data points with counts greater than or equal to the threshold. We then selected common features that had a minimum length of 10 ms, regardless of whether they were consecutive or not. Next, we looked for points in the

selected common features that covered at least 25 ms, with at most 25 missed points in between, to ensure a robust and meaningful analysis.

Software and hardware

We employed the EEGLAB toolbox (Delorme & Makeig, 2004) for preprocessing EEG data and utilised the SPM, version 12 (SPM12) toolbox in MATLAB (Version 2022b) for analysing fMRI data, DCM estimations, and statistical analyses For clustering RTs, we utilised IBM SPSS Statistics (Version 27) on a local Windows machine equipped with 14 CPU cores, Intel i9-13900H, and 64GB RAM. All other data analyses were conducted on the Northern Ireland High Performance Computing (NI-HPC) facility using the Kelvin2 system (www.ni-hpc.ac.uk).

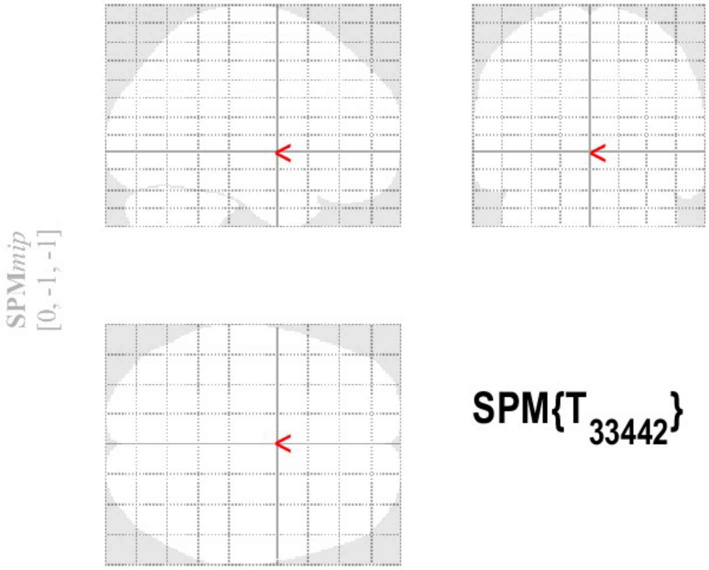

SPM{T_{33442}}

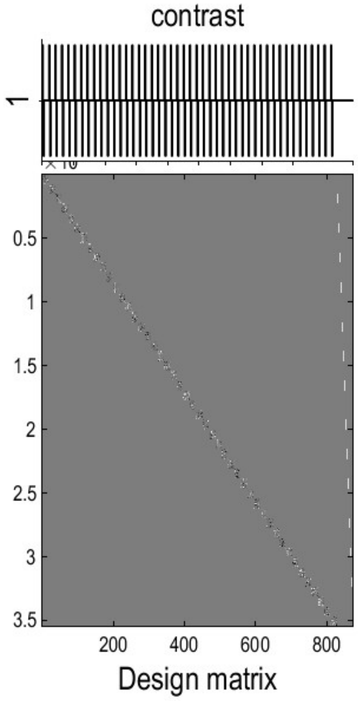

SPMresults: .\Second Level Stats
Height threshold T = 4.471160 {p<0.05 (FWE)}
Extent threshold k = 0 voxels

Statistics: ***p-values adjusted for search volume***

set-level		cluster-level				peak-level					mm mm mm
p	c	$p_{FWE-corr}$	$q_{FDR-corr}$	k_E	p_{uncorr}	$p_{FWE-corr}$	$q_{FDR-corr}$	T	(Z_E)	p_{uncorr}	

no suprathreshold clusters

table shows 3 local maxima more than 8.0mm apart

Height threshold: T = 4.47, p = 0.000 (0.050)
Extent threshold: k = 0 voxels
Expected voxels per cluster, <k> = 2.836
Expected number of clusters, <c> = 0.05
FWEp: 4.471, FDRp: Inf, FWEc: Inf, FDRc: Inf

Degrees of freedom = [1.0, 33442.0]
FWHM = 12.3 12.5 13.0 mm mm mm; 4.1 4.2 4.3 {voxels}
Volume: 939924 = 34812 voxels = 404.6 resels
Voxel size: 3.0 3.0 3.0 mm mm mm; (resel = 73.84 voxels)

Fig. S1. Statistical parametric mapping (SPM) analysis demonstrating non-significant brain region activation. The SPM{T} maps depict the lack of suprathreshold clusters when contrasting low confidence against high confidence sessions, across all sessions during the stimulation/response phase. Thresholds were set at a height of $T = 4.471160$, $p < 0.05$ (family-wise error corrected), and an extent threshold of $k = 0$ voxels, indicating no significant activation. The design matrix and contrast vector are provided, alongside the adjusted p-values for search volume, which confirm the absence of significant differences. The table below the SPM{T} maps specify that there are no local maxima surpassing the 8.0 mm separation threshold, further supporting the null findings.

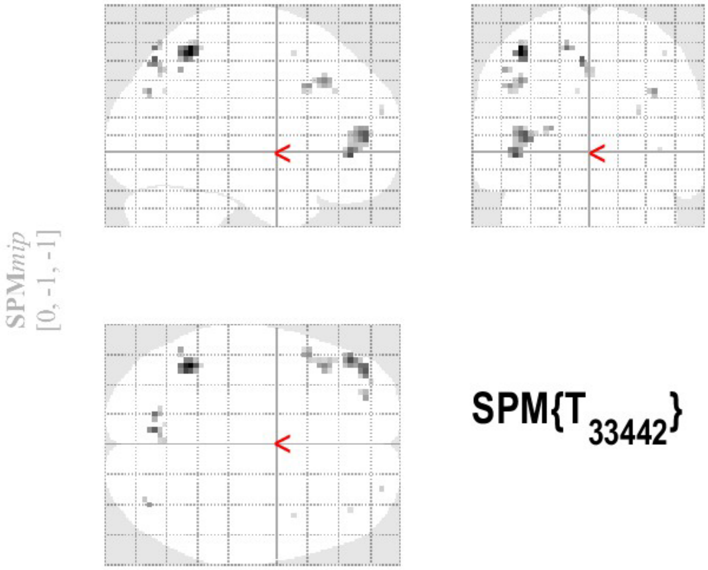

SPM{T_{33442}}

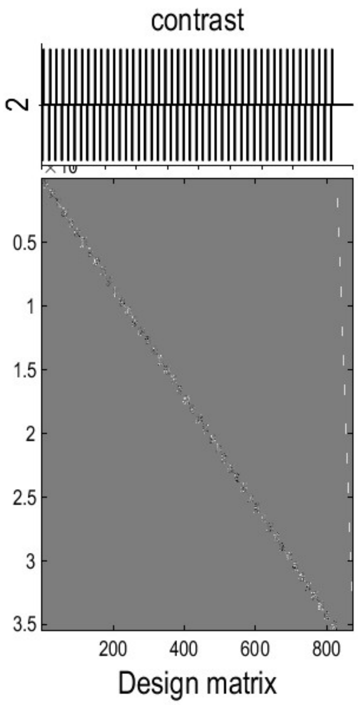

SPMresults: .\Second Level Stats
Height threshold T = 4.471160 {p<0.05 (FWE)}
Extent threshold k = 0 voxels

Statistics: *p-values adjusted for search volume*

set-level		cluster-level				peak-level					mm mm mm		
p	c	$p_{FWE-corr}$	$q_{FDR-corr}$	k_E	p_{uncorr}	$p_{FWE-corr}$	$q_{FDR-corr}$	T	(Z_E)	p_{uncorr}			
0.000	12	0.000	0.030	26	0.005	0.000	0.110	5.53	5.53	0.000	-42	-52	56
		0.000	0.007	43	0.001	0.003	0.254	5.15	5.15	0.000	-45	41	-4
						0.003	0.254	5.09	5.09	0.000	-39	50	8
		0.004	0.220	9	0.073	0.003	0.254	5.09	5.08	0.000	-6	-73	50
		0.009	0.411	5	0.171	0.005	0.321	4.99	4.99	0.000	-15	-73	56
		0.000	0.035	22	0.009	0.009	0.438	4.87	4.87	0.000	-42	26	38
						0.011	0.438	4.83	4.83	0.000	-48	17	35
		0.015	0.489	3	0.285	0.012	0.438	4.81	4.81	0.000	36	-76	32
		0.015	0.489	3	0.285	0.015	0.497	4.76	4.76	0.000	-24	50	11
		0.019	0.512	2	0.384	0.017	0.497	4.73	4.73	0.000	-51	-58	44
		0.019	0.512	2	0.384	0.037	0.911	4.55	4.55	0.000	27	59	20
		0.028	0.547	1	0.547	0.044	0.969	4.51	4.51	0.000	42	8	53
		0.028	0.547	1	0.547	0.048	0.969	4.48	4.48	0.000	-9	-67	32
		0.028	0.547	1	0.547	0.048	0.969	4.48	4.48	0.000	39	41	-1

table shows 3 local maxima more than 8.0mm apart

Height threshold: T = 4.47, p = 0.000 (0.050)
Extent threshold: k = 0 voxels
Expected voxels per cluster, <k> = 2.836
Expected number of clusters, <c> = 0.05
FWEp: 4.471, FDRp: Inf, FWEc: 1, FDRc: 22

Degrees of freedom = [1.0, 33442.0]
FWHM = 12.3 12.5 13.0 mm mm mm; 4.1 4.2 4.3 {voxels}
Volume: 939924 = 34812 voxels = 404.6 resels
Voxel size: 3.0 3.0 3.0 mm mm mm; (resel = 73.84 voxels)

Fig. S2. Significant brain activation during stimulation/response phase in high-vs-low confidence rating condition. SPM results illustrate marked activation in the IPL (left hemisphere), MFG (both left and right hemispheres), PreCUN (left hemisphere), SPL (left hemisphere), Angular Gyrus (right hemisphere), and SFG (both left and right hemispheres). These findings are indicative of varied neural involvement correlating with confidence levels during task performance when high confidence sessions are more active than low confidence sessions. The fMRI SPM{T} analysis parallels the thresholds and statistical parameters detailed in Fig. S1. The accompanying results table confirms the presence of three distinct local maxima separated by more than 8.0 mm, validating the significant clusters identified.

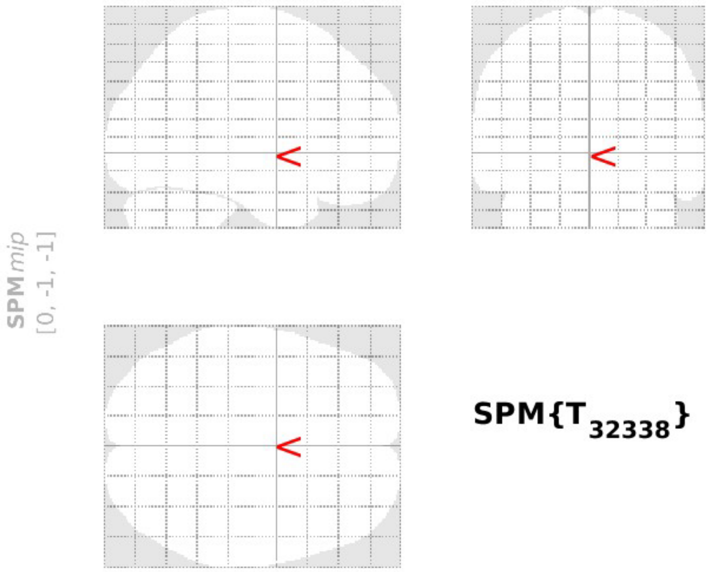

SPM{T_{32338}}

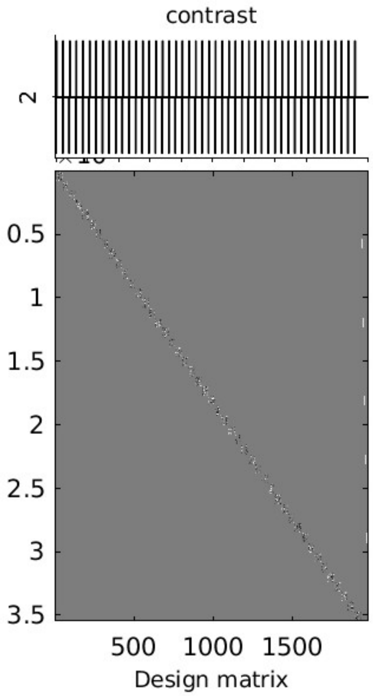

SPMresults: ..igh conditions in all phases
Height threshold T = 4.475290 {p<0.05 (FWE)}
Extent threshold k = 0 voxels

Statistics: ***p-values adjusted for search volume***

set-level		cluster-level				peak-level					mm mm mm
p	c	$p_{FWE-corr}$	$q_{FDR-corr}$	k_E	p_{uncorr}	$p_{FWE-corr}$	$q_{FDR-corr}$	T	(Z_E)	p_{uncorr}	

no suprathreshold clusters

table shows 3 local maxima more than 8.0mm apart

Height threshold: T = 4.48, p = 0.000 (0.050)
Extent threshold: k = 0 voxels
Expected voxels per cluster, <k> = 2.779
Expected number of clusters, <c> = 0.05
FWEp: 4.475, FDRp: Inf, FWEc: Inf, FDRc: Inf

Degrees of freedom = [1.0, 32338.0]
FWHM = 12.2 12.4 12.9 mm mm mm; 4.1 4.1 4.3 {voxels}
Volume: 939924 = 34812 voxels = 411.8 resels
Voxel size: 3.0 3.0 3.0 mm mm mm; (resel = 72.54 voxels)

Fig. S3. No significant differential brain activation during stimulation/response phase in high-vs-low confidence rating condition when excluding subjects with non-significant RT-confidence relationship. The fMRI SPM{T} analysis parallels the thresholds and statistical parameters detailed in Fig. S1, with results indicating a lack of significant activation differences.

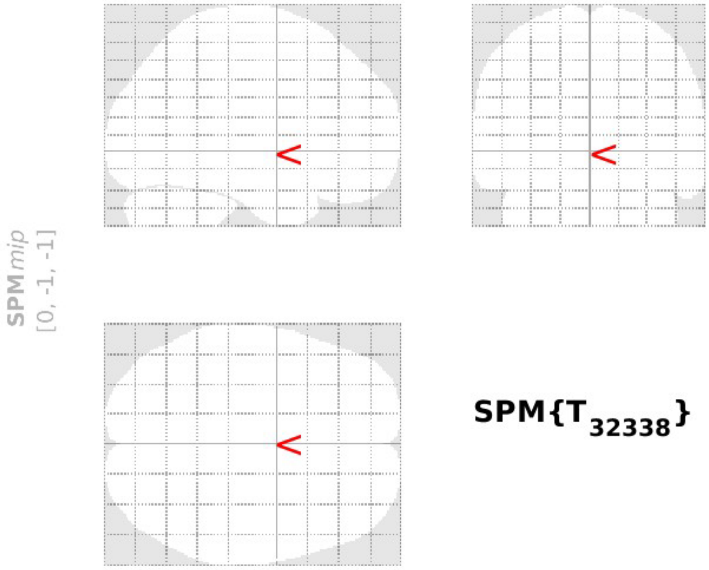

SPM{T$_{32338}$}

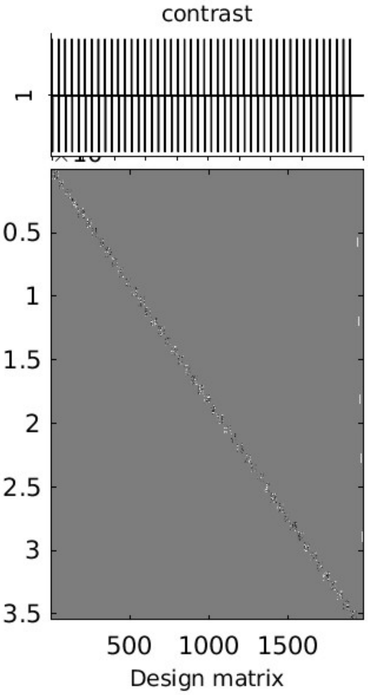

SPMresults: ..igh conditions in all phases
Height threshold T = 4.475290 {p<0.05 (FWE)}
Extent threshold k = 0 voxels

Statistics: ***p-values adjusted for search volume***

set-level		cluster-level				peak-level					mm mm mm
p	*c*	$p_{FWE-corr}$	$q_{FDR-corr}$	k_E	p_{uncorr}	$p_{FWE-corr}$	$q_{FDR-corr}$	*T*	(Z_E)	p_{uncorr}	

no suprathreshold clusters

table shows 3 local maxima more than 8.0mm apart

Height threshold: T = 4.48, p = 0.000 (0.050)
Extent threshold: k = 0 voxels
Expected voxels per cluster, <k> = 2.779
Expected number of clusters, <c> = 0.05
FWEp: 4.475, FDRp: Inf, FWEc: Inf, FDRc: Inf

Degrees of freedom = [1.0, 32338.0]
FWHM = 12.2 12.4 12.9 mm mm mm; 4.1 4.1 4.3 {voxels}
Volume: 939924 = 34812 voxels = 411.8 resels
Voxel size: 3.0 3.0 3.0 mm mm mm; (resel = 72.54 voxels)

Fig. S4. No significant differential brain activation during stimulation/response phase in low-vs-high confidence rating condition when excluding subjects with non-significant RT-confidence relationship. The fMRI SPM{T} analysis parallels the thresholds and statistical parameters detailed in Fig. S1, with results indicating a lack of significant activation differences.

SPM*mip*
[0, -1, -1]

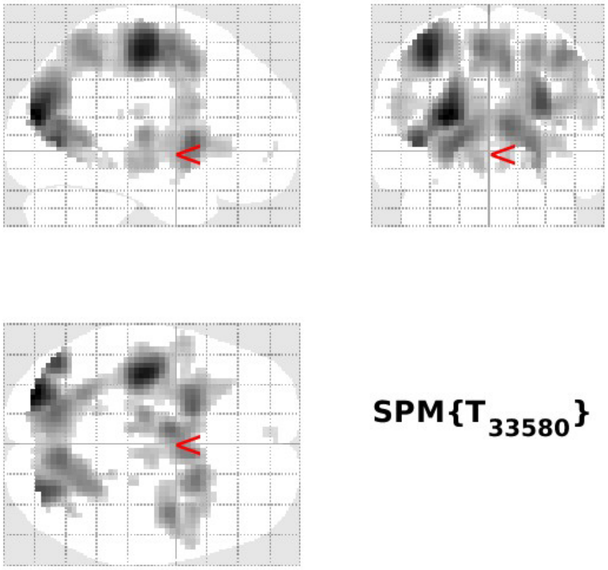

SPM{T_{33580}}

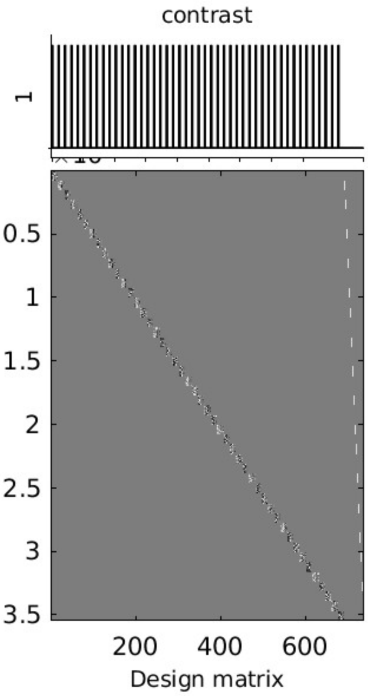

SPMresults: .. Stats for stimulation phase
Height threshold T = 4.474987 {p<0.05 (FWE)}
Extent threshold k = 0 voxels

Statistics: *p-values adjusted for search volume*

set-level		cluster-level				peak-level					
p	*c*	$p_{FWE-corr}$	$q_{FDR-corr}$	k_E	p_{uncorr}	$p_{FWE-corr}$	$q_{FDR-corr}$	*T*	(Z_E)	p_{uncorr}	mm mm mm
**0.000**	**13**	**0.000**	**0.000**	**2796**	**0.000**	**0.000**	**0.000**	**14.39**	**Inf**	**0.000**	**-24 -85 20**
						0.000	0.000	13.20	Inf	0.000	-36 -22 56
						0.000	0.000	11.12	Inf	0.000	-45 -70 2
		**0.000**	**0.000**	**402**	**0.000**	**0.000**	**0.000**	**10.15**	**Inf**	**0.000**	**-21 8 2**
						0.000	0.001	6.01	6.01	0.000	-30 26 -1
		**0.000**	**0.000**	**498**	**0.000**	**0.000**	**0.000**	**9.62**	**Inf**	**0.000**	**-6 -4 56**
						0.000	0.000	8.76	Inf	0.000	9 8 47
						0.000	0.000	8.63	Inf	0.000	6 2 53
		**0.000**	**0.000**	**242**	**0.000**	**0.000**	**0.000**	**9.15**	**Inf**	**0.000**	**21 11 2**
						0.000	0.000	8.41	Inf	0.000	27 11 -4
						0.000	0.000	6.12	6.12	0.000	36 14 2
		**0.000**	**0.000**	**415**	**0.000**	**0.000**	**0.000**	**8.88**	**Inf**	**0.000**	**42 -7 50**
						0.000	0.000	7.17	7.16	0.000	39 8 23
						0.000	0.001	6.02	6.02	0.000	48 2 38
		**0.000**	**0.000**	**212**	**0.000**	**0.000**	**0.000**	**7.95**	**Inf**	**0.000**	**-15 -19 8**
						0.000	0.000	6.75	6.74	0.000	-9 -19 -7
						0.001	0.030	5.29	5.29	0.000	-21 -28 -4
		**0.000**	**0.000**	**113**	**0.000**	**0.000**	**0.000**	**6.15**	**6.15**	**0.000**	**9 -16 -7**
						0.000	0.000	6.13	6.13	0.000	9 -16 5
		**0.001**	**0.021**	**19**	**0.013**	**0.000**	**0.010**	**5.52**	**5.52**	**0.000**	**-51 -25 20**
		**0.005**	**0.139**	**7**	**0.107**	**0.001**	**0.022**	**5.36**	**5.36**	**0.000**	**24 -25 -7**
		**0.019**	**0.411**	**2**	**0.379**	**0.010**	**0.217**	**4.86**	**4.86**	**0.000**	**-12 -25 38**
		**0.014**	**0.331**	**3**	**0.280**	**0.014**	**0.284**	**4.79**	**4.78**	**0.000**	**-36 -34 17**
		**0.004**	**0.125**	**8**	**0.087**	**0.018**	**0.368**	**4.72**	**4.72**	**0.000**	**-6 53 -4**
		**0.027**	**0.543**	**1**	**0.543**	**0.039**	**0.786**	**4.53**	**4.53**	**0.000**	**-33 -4 -19**

table shows 3 local maxima more than 8.0mm apart

Height threshold: T = 4.47, p = 0.000 (0.050)
Extent threshold: k = 0 voxels
Expected voxels per cluster, <k> = 2.782
Expected number of clusters, <c> = 0.05
FWEp: 4.475, FDRp: 5.292, FWEc: 1, FDRc: 19

Degrees of freedom = [1.0, 33580.0]
FWHM = 12.2 12.4 12.9 mm mm mm; 4.1 4.1 4.3 {voxels}
Volume: 939924 = 34812 voxels = 411.4 resels
Voxel size: 3.0 3.0 3.0 mm mm mm; (resel = 72.62 voxels)

Fig. S5. Significant sensory and movement related active regions against implicit baseline during stimulation phase. The fMRI SPM{T} analysis parallels the thresholds and statistical parameters detailed in Fig. S1. Active brain regions are in occipital, parietal, and frontal regions. The extracted areas include undesired regions related to movements, stimulus processing, and other non-relevant brain activities which could obscure the ability to extract key regions specifically relevant to confidence or choice-based RT.

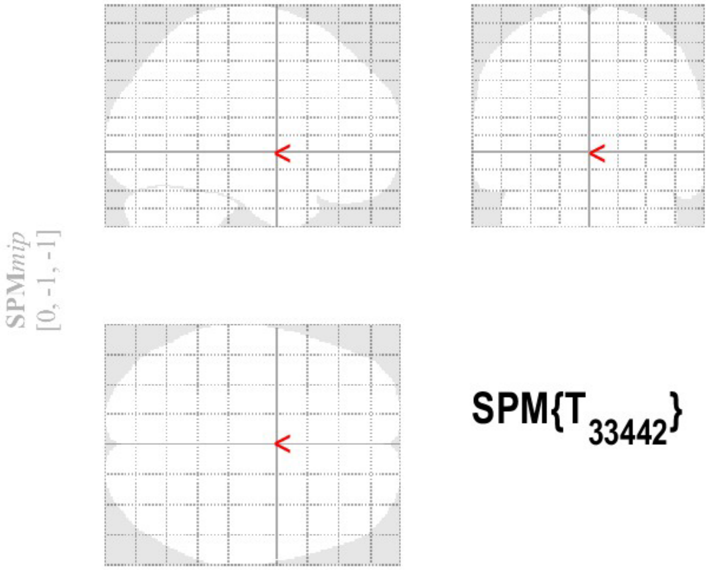

SPM{T_{33442}}

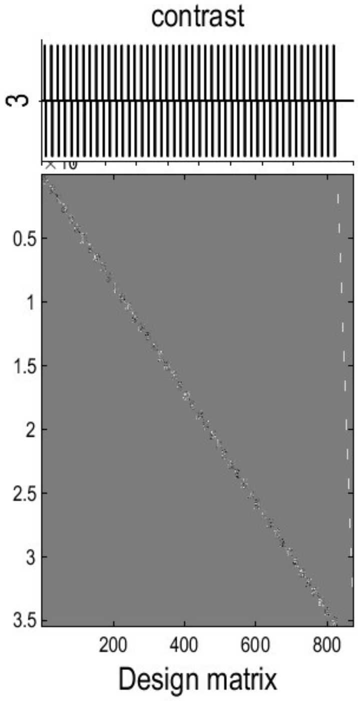

SPMresults: .\Second Level Stats
Height threshold T = 4.471160 {p<0.05 (FWE)}
Extent threshold k = 0 voxels

Statistics: ***p-values adjusted for search volume***

set-level		cluster-level				peak-level					mm mm mm
p	c	$p_{FWE-corr}$	$q_{FDR-corr}$	k_E	p_{uncorr}	$p_{FWE-corr}$	$q_{FDR-corr}$	T	(Z_E)	p_{uncorr}	

no suprathreshold clusters

table shows 3 local maxima more than 8.0mm apart

Height threshold: T = 4.47, p = 0.000 (0.050)
Extent threshold: k = 0 voxels
Expected voxels per cluster, <k> = 2.836
Expected number of clusters, <c> = 0.05
FWEp: 4.471, FDRp: Inf, FWEc: Inf, FDRc: Inf

Degrees of freedom = [1.0, 33442.0]
FWHM = 12.3 12.5 13.0 mm mm mm; 4.1 4.2 4.3 {voxels}
Volume: 939924 = 34812 voxels = 404.6 resels
Voxel size: 3.0 3.0 3.0 mm mm mm; (resel = 73.84 voxels)

Fig. S6. No significant differential brain activation between correct and incorrect responses during the stimulation/response phase. The fMRI SPM{T} analysis parallels the thresholds and statistical parameters detailed in Fig. S1, with results indicating a lack of significant activation differences. The data affirm the consistency of brain activity irrespective of response accuracy.

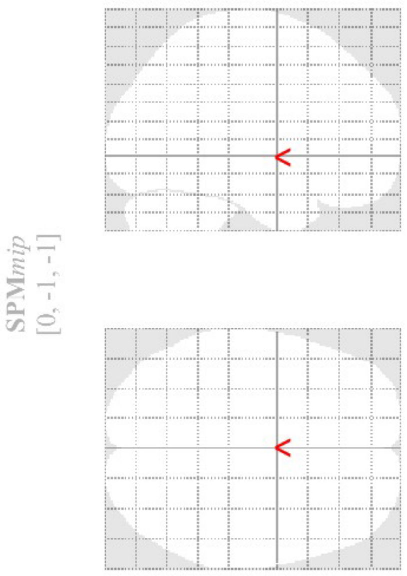

SPM{T_{33442}}

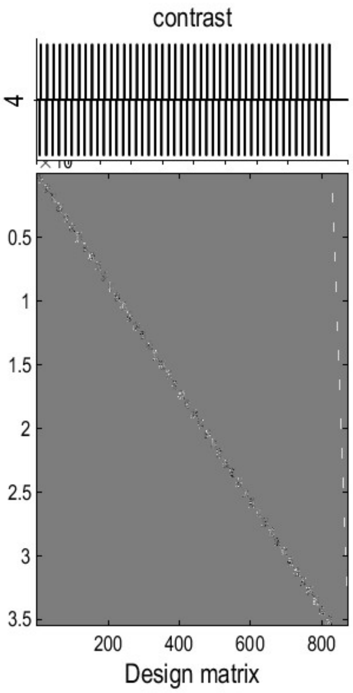

SPMresults: .\Second Level Stats
Height threshold T = 4.471160 {p<0.05 (FWE)}
Extent threshold k = 0 voxels

Statistics: *p-values adjusted for search volume*

set-level		cluster-level				peak-level					mm mm mm
p	c	$p_{FWE\text{-}corr}$	$q_{FDR\text{-}corr}$	k_E	p_{uncorr}	$p_{FWE\text{-}corr}$	$q_{FDR\text{-}corr}$	T	(Z_E)	p_{uncorr}	

no suprathreshold clusters

table shows 3 local maxima more than 8.0mm apart

Height threshold: T = 4.47, p = 0.000 (0.050)
Extent threshold: k = 0 voxels
Expected voxels per cluster, <k> = 2.836
Expected number of clusters, <c> = 0.05
FWEp: 4.471, FDRp: Inf, FWEc: Inf, FDRc: Inf

Degrees of freedom = [1.0, 33442.0]
FWHM = 12.3 12.5 13.0 mm mm mm; 4.1 4.2 4.3 {voxels}
Volume: 939924 = 34812 voxels = 404.6 resels
Voxel size: 3.0 3.0 3.0 mm mm mm; (resel = 73.84 voxels)

Fig. S7. No significant differential brain activation when comparing incorrect to correct choices during the stimulation/response phase. This fMRI SPM{T} analysis adheres to the thresholds and statistical parameters specified in Fig. S1's caption, and similarly, it reveals no significant activation differences.

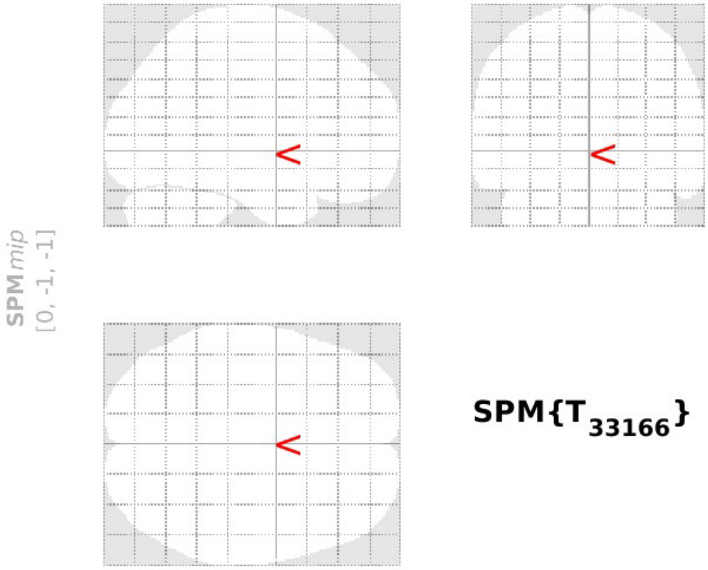

SPM{T_{33166}}

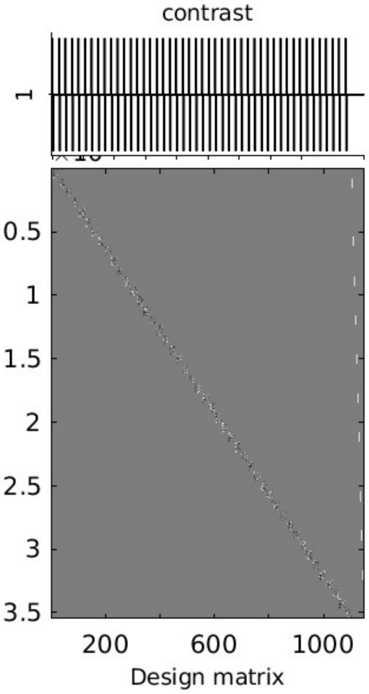

SPMresults: ..ect conditions in all phases
Height threshold T = 4.471866 {p<0.05 (FWE)}
Extent threshold k = 0 voxels

Statistics: ***p-values adjusted for search volume***

set-level		cluster-level				peak-level					mm mm mm
p	c	$p_{FWE-corr}$	$q_{FDR-corr}$	k_E	p_{uncorr}	$p_{FWE-corr}$	$q_{FDR-corr}$	T	(Z_E)	p_{uncorr}	

no suprathreshold clusters

table shows 3 local maxima more than 8.0mm apart

Height threshold: T = 4.47, p = 0.000 (0.050)
Extent threshold: k = 0 voxels
Expected voxels per cluster, <k> = 2.826
Expected number of clusters, <c> = 0.05
FWEp: 4.472, FDRp: Inf, FWEc: Inf, FDRc: Inf

Degrees of freedom = [1.0, 33166.0]
FWHM = 12.3 12.5 13.0 mm mm mm; 4.1 4.2 4.3 {voxels}
Volume: 939924 = 34812 voxels = 405.8 resels
Voxel size: 3.0 3.0 3.0 mm mm mm; (resel = 73.62 voxels)

Fig. S8. No significant differential brain activity for fMRI SPM{T} analysis for leftward-vs-rightward stimulus condition during stimulation/response phase. This indicates a lack of differential brain activity between the directions of stimuli. For statistical thresholds and parameters, refer to Fig. S1. The results suggest that the directionality of stimulus during the stimulation phase does not elicit a distinct neural response pattern.

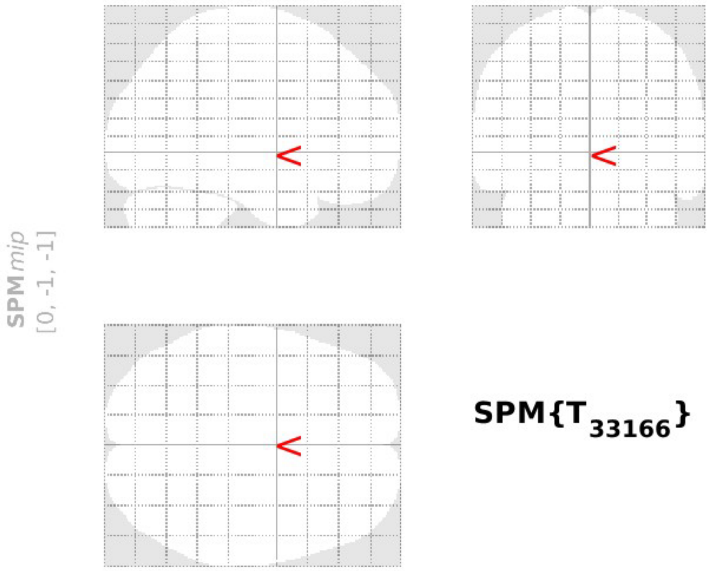

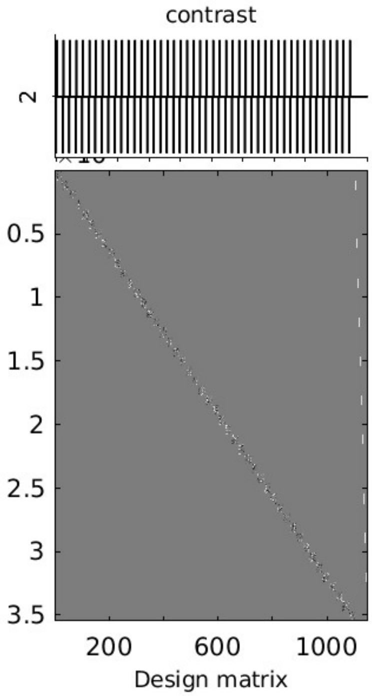

SPMresults: ..ect conditions in all phases
Height threshold T = 4.471866 {p<0.05 (FWE)}
Extent threshold k = 0 voxels

Statistics: ***p-values adjusted for search volume***

set-level		cluster-level				peak-level					mm mm mm
p	c	$p_{FWE-corr}$	$q_{FDR-corr}$	k_E	p_{uncorr}	$p_{FWE-corr}$	$q_{FDR-corr}$	T	(Z_E)	p_{uncorr}	

no suprathreshold clusters

table shows 3 local maxima more than 8.0mm apart

Height threshold: T = 4.47, p = 0.000 (0.050)
Extent threshold: k = 0 voxels
Expected voxels per cluster, <k> = 2.826
Expected number of clusters, <c> = 0.05
FWEp: 4.472, FDRp: Inf, FWEc: Inf, FDRc: Inf

Degrees of freedom = [1.0, 33166.0]
FWHM = 12.3 12.5 13.0 mm mm mm; 4.1 4.2 4.3 {voxels}
Volume: 939924 = 34812 voxels = 405.8 resels
Voxel size: 3.0 3.0 3.0 mm mm mm; (resel = 73.62 voxels)

Fig. S9. No significant differential brain activity for fMRI SPM{T} analysis for rightward-vs-leftward stimulus condition during stimulation/response phase. No suprathreshold clusters were observed, suggesting similar neural engagement for stimuli directionality. Refer to the statistical details in Fig. S1 for comparison. These findings indicate that the direction of stimulus in the stimulation phase is not a differentiating factor for neural activation.

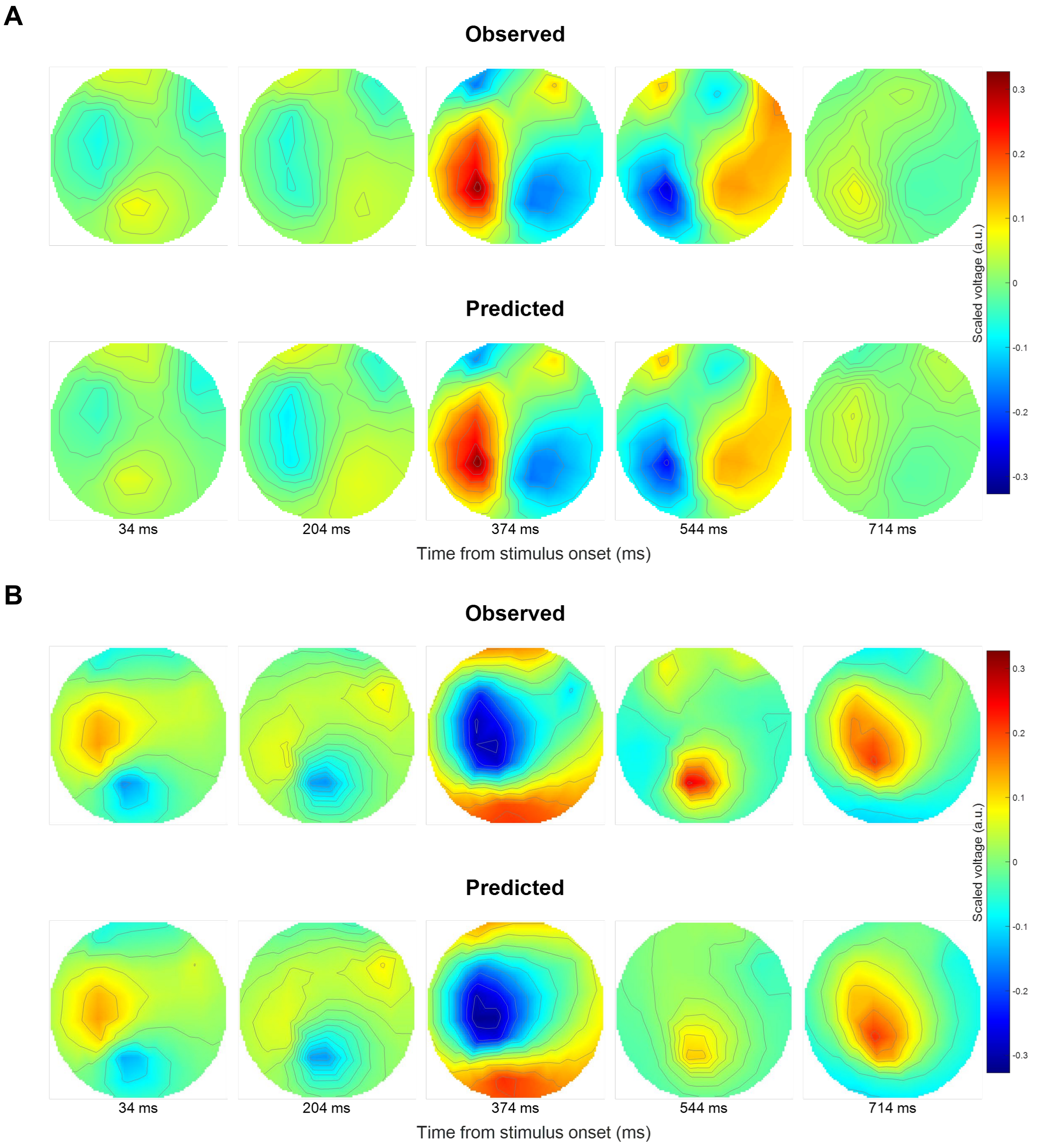

Fig. S10. Scalp topography depicting differential EEG activity patterns for high versus low confidence trials in the most accurately modelled participant, as per the fMRI-informed EEG-DCM analysis. (A) EEG scalp activity for high confidence trials, where the winning fMRI-informed EEG- Dynamic causal modelling (DCM) model for the best-fitted participant indicates a left-hemispheric positivity and right-hemispheric negativity at 374 ms post-stimulus. This activity pattern then reverses into left-hemisphere negativity and right-hemisphere positivity by 544 ms. (B) low confidence trials, with centro-parietal negativity at 370 ms evolving into a widespread positivity by 714 ms. These maps align closely with recorded EEG scalp distributions, validating the predictive capability of the EEG-DCM model in capturing the spatial-temporal dynamics of neural activity associated with confidence levels.

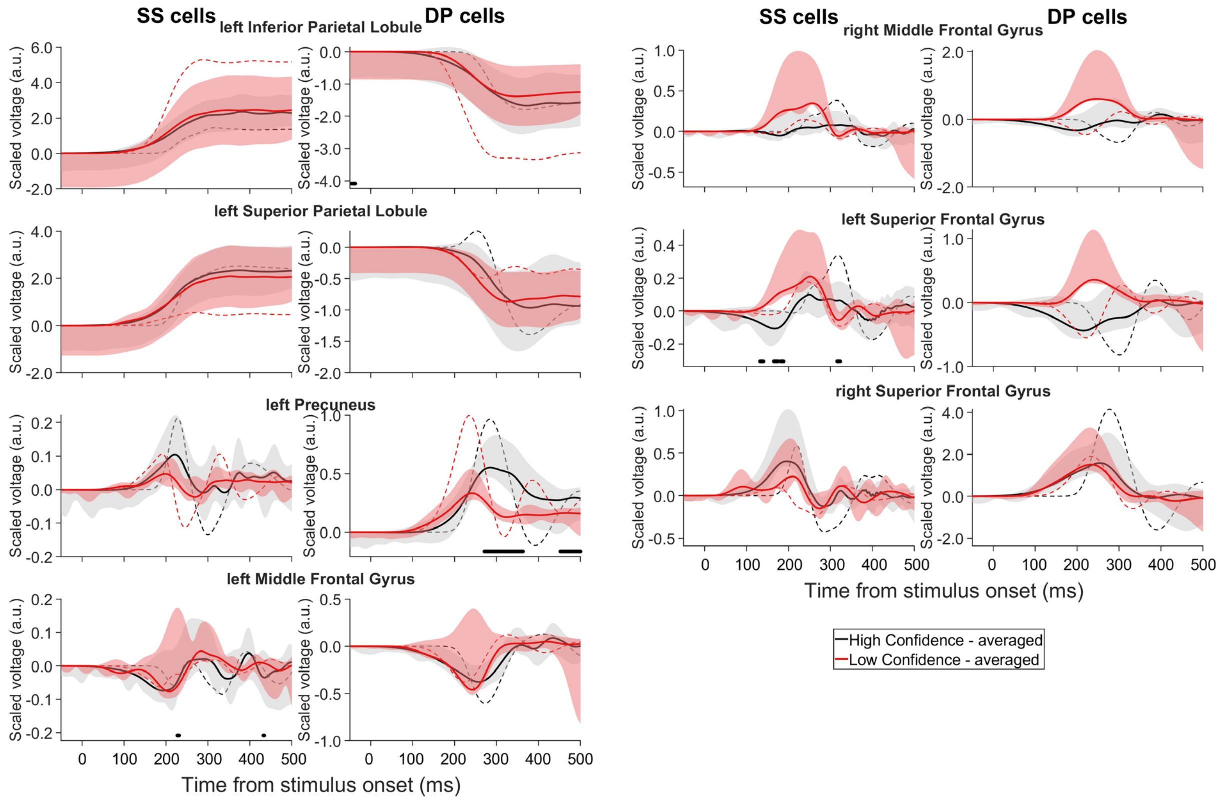

Fig. S11. Predominant encoding of confidence rating by DP activity in left PreCUN and potential encoding of subjective uncertainty by SS activity in left SFG. Estimated scaled voltage for SS neural population and scaled current for DP neural populations from the model that was deemed most accurate. Black (red) lines: High (low) confidence ratings. Solid (dashed) lines: averages across participants (best-fitted participant). Shaded areas: 95% confidence. Filled markers above horizontal axis: time points where significant differences were found between the averaged conditions ($p < 0.05$). Left SFG shows increased SS activity during low confidence trials, suggesting encoding of decision uncertainty, whereas left PreCUN exhibits greater DP activity during high confidence trials, indicating encoding of subjective confidence.

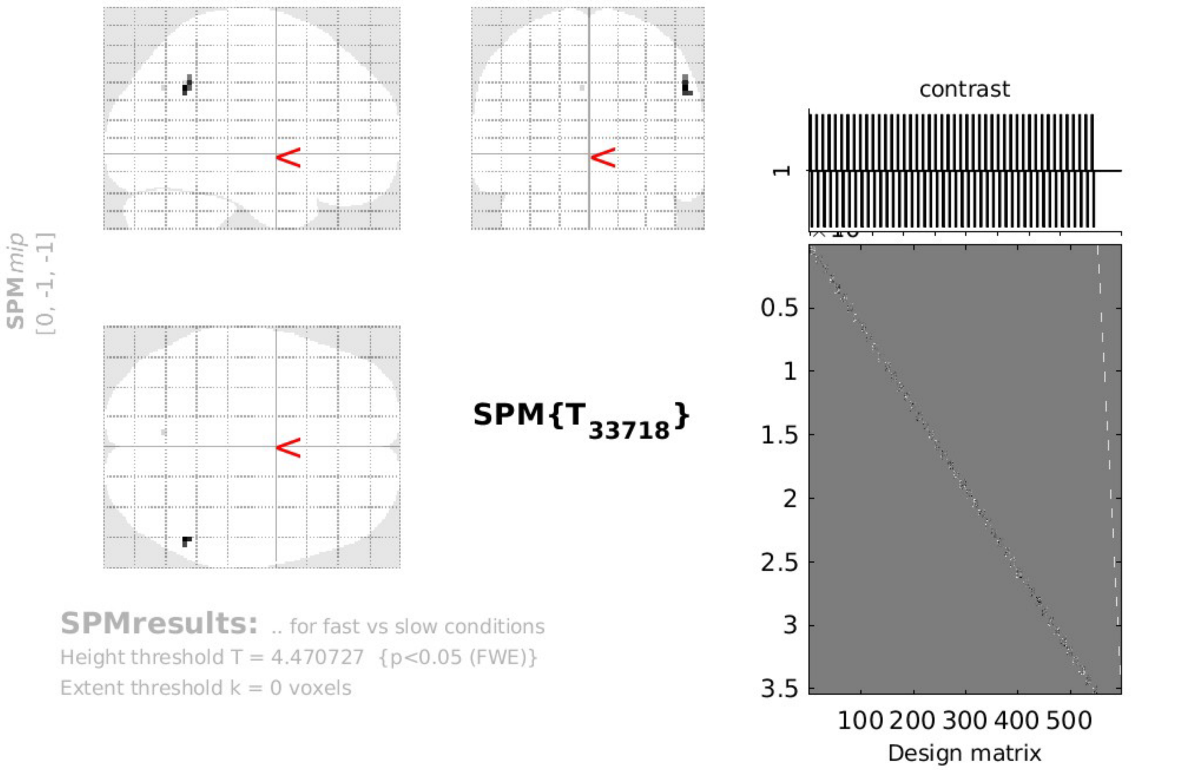

Statistics: ***p-values adjusted for search volume***

set-level		cluster-level				peak-level					mm mm mm
p	c	$p_{FWE-corr}$	$q_{FDR-corr}$	k_E	p_{uncorr}	$p_{FWE-corr}$	$q_{FDR-corr}$	T	(Z_E)	p_{uncorr}	
0.001	2	0.006	0.221	7	0.110	0.014	0.566	4.77	4.77	0.000	54 -55 35
		0.028	0.547	1	0.547	0.042	0.834	4.52	4.51	0.000	-6 -67 35

table shows 3 local maxima more than 8.0mm apart

Height threshold: T = 4.47, p = 0.000 (0.050)
Extent threshold: k = 0 voxels
Expected voxels per cluster, <k> = 2.843
Expected number of clusters, <c> = 0.05
FWEp: 4.471, FDRp: Inf, FWEc: 1, FDRc: Inf

Degrees of freedom = [1.0, 33718.0]
FWHM = 12.3 12.5 13.0 mm mm mm; 4.1 4.2 4.3 {voxels}
Volume: 939924 = 34812 voxels = 403.8 resels
Voxel size: 3.0 3.0 3.0 mm mm mm; (resel = 73.98 voxels)

Fig. S12. Higher brain activation associated with fast-vs-slow choice-based RT condition. SMG in right parietal lobe and the PreCUN in left parietal lobe were significantly more active during fast RTs compared to slow RTs. This pattern of activation correlates with the efficiency of response execution. For detailed statistical thresholds and parameters, see Fig. S1 caption.

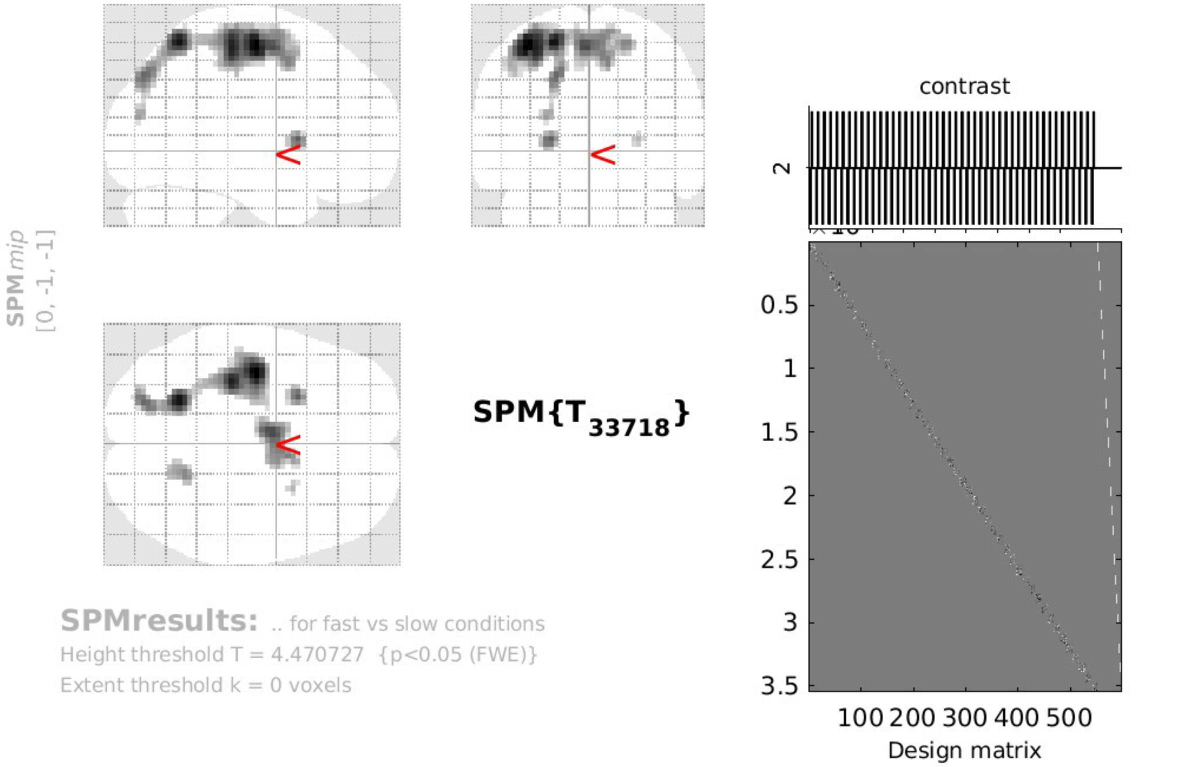

Statistics: ***p-values adjusted for search volume***

set-level		cluster-level				peak-level					mm mm mm
p	c	$p_{FWE-corr}$	$q_{FDR-corr}$	k_E	p_{uncorr}	$p_{FWE-corr}$	$q_{FDR-corr}$	T	(Z_E)	p_{uncorr}	
**0.000**	**5**	**0.000**	**0.000**	**638**	**0.000**	**0.000**	**0.000**	**7.87**	**Inf**	**0.000**	**-39 -13 56**
						0.000	0.000	7.48	7.48	0.000	-21 -58 59
						0.000	0.000	7.33	7.33	0.000	-33 -28 56
		**0.000**	**0.000**	**238**	**0.000**	**0.000**	**0.000**	**6.66**	**6.66**	**0.000**	**-3 -4 56**
						0.000	0.002	5.88	5.88	0.000	6 2 53
		**0.000**	**0.001**	**39**	**0.001**	**0.000**	**0.000**	**6.33**	**6.33**	**0.000**	**-24 11 5**
		**0.000**	**0.000**	**53**	**0.000**	**0.000**	**0.003**	**5.77**	**5.77**	**0.000**	**18 -55 59**
		**0.004**	**0.074**	**9**	**0.074**	**0.004**	**0.070**	**5.08**	**5.08**	**0.000**	**27 8 5**

table shows 3 local maxima more than 8.0mm apart

Height threshold: T = 4.47, p = 0.000 (0.050)
Extent threshold: k = 0 voxels
Expected voxels per cluster, <k> = 2.843
Expected number of clusters, <c> = 0.05
FWEp: 4.471, FDRp: 5.466, FWEc: 9, FDRc: 39

Degrees of freedom = [1.0, 33718.0]
FWHM = 12.3 12.5 13.0 mm mm mm; 4.1 4.2 4.3 {voxels}
Volume: 939924 = 34812 voxels = 403.8 resels
Voxel size: 3.0 3.0 3.0 mm mm mm; (resel = 73.98 voxels)

Fig. S13. Significantly higher brain activity in specific regions for slow-vs-fast choice-based RT condition. The regions include left PreCG, left medial frontal gyrus, left lentiform nucleus (sub-lobar), right SPL, and right extra-nuclear area (sub-lobar). Refer to Fig. S1 for statistical thresholds and parameters. This pattern suggests that slower RTs are associated with increased neural activities in areas implicated in motor control, cognitive processing, and sensory integration.

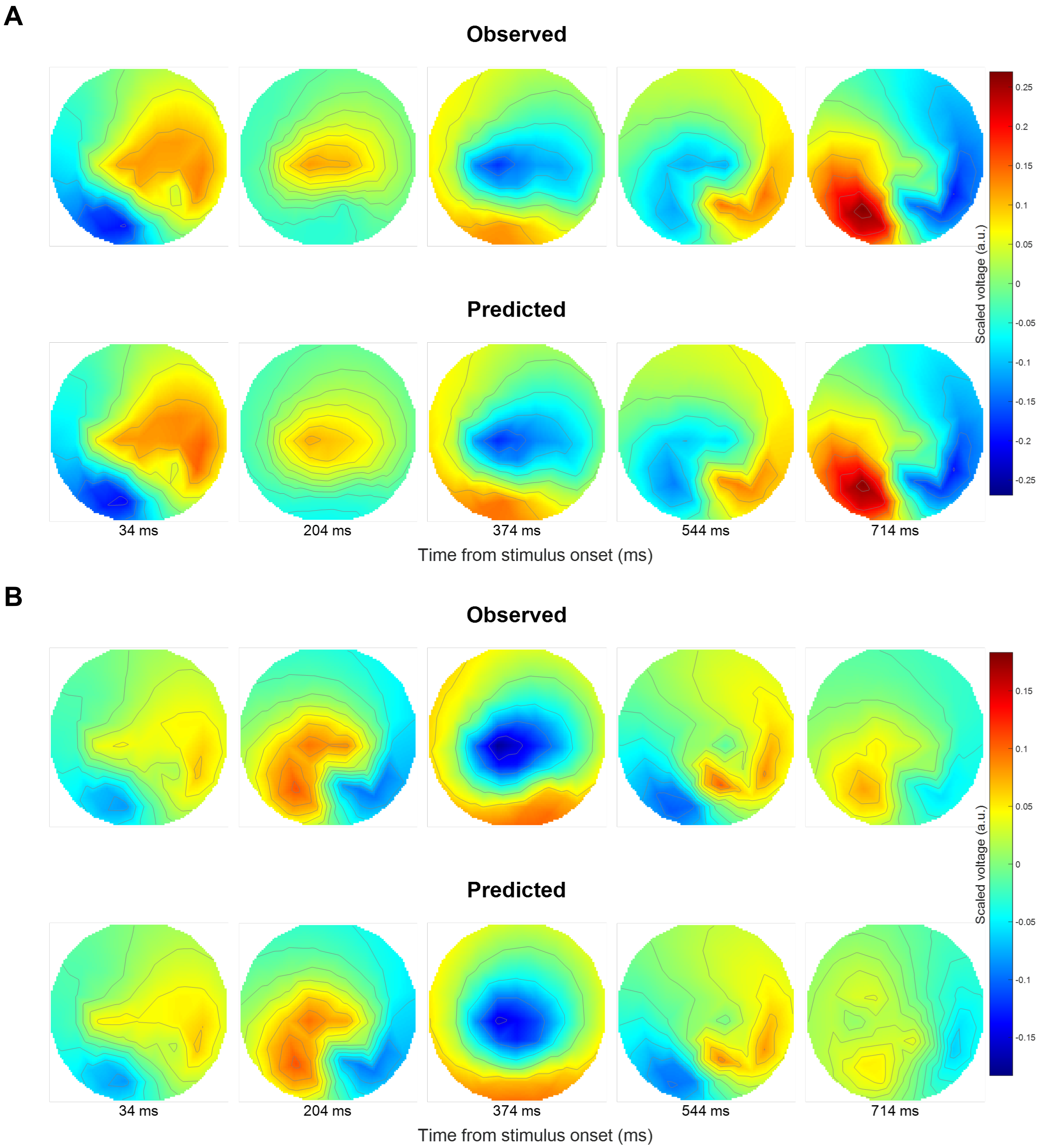

Fig. S14. Scalp maps representing observed and model-predicted EEG activity during the stimulation/response phase for trials categorized by choice-based RTs. (A) Trials with fast RT, showing a centro-parietal negativity at 370 ms post-stimulus onset, transitioning to a positivity in the left hemisphere and negativity in the right hemisphere at 700 ms. (B) Trials with slow RT, where initial centro-parietal negativity at 370 ms is followed by a left-hemispheric negativity and a right-hemispheric positivity by 544 ms. These patterns indicate a clear distinction in neural processing speed and lateralization associated with the speed of participants' responses. The consistency of topographical distribution between predicted and observed EEG activity underscores the precision of the EEG-DCM model in capturing the temporal dynamics of neural responses related to RTs.

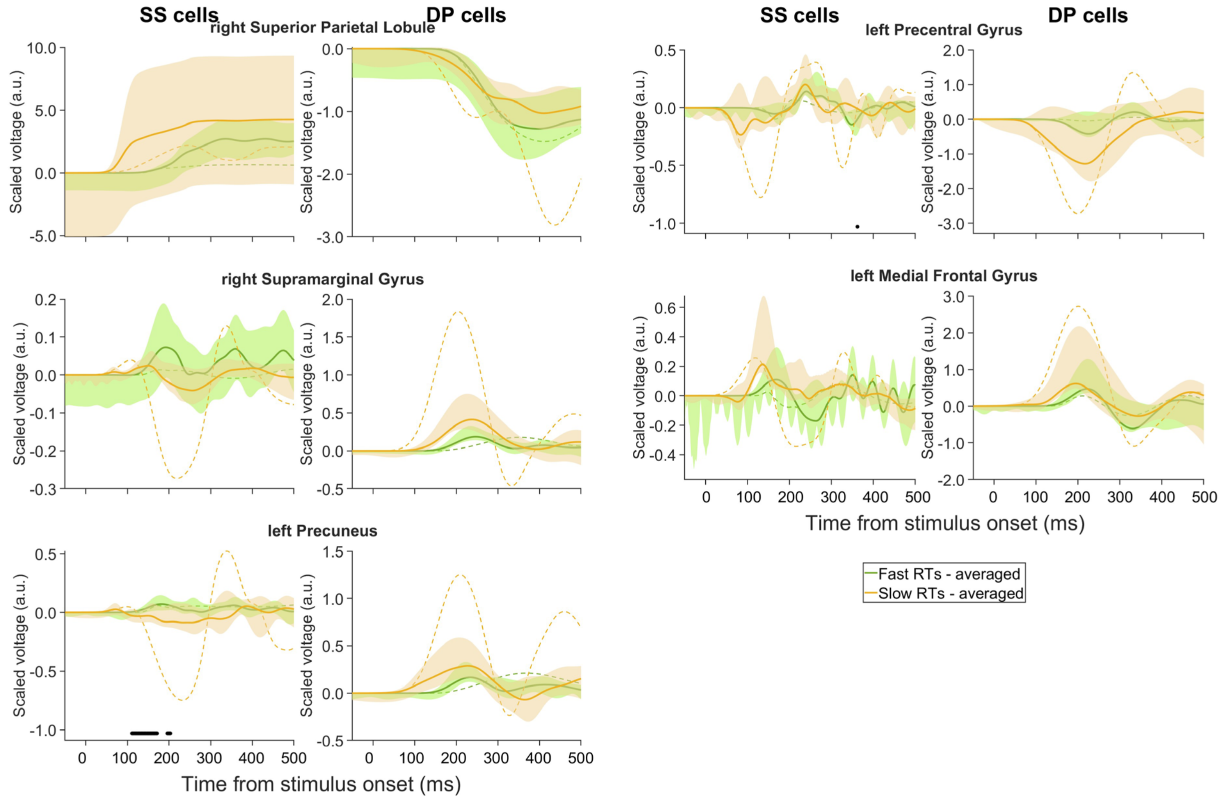

Fig. S15. Involvement of left PreCUN in encoding fast RT, suggesting objective evaluation of decision confidence. Estimated scaled voltage for SS neural populations and scaled current for DP neural populations in the winning model, with fast choice-based RTs (green) and slow choice-based RTs (orange). Across most regions, SS activity is consistent between slow and fast choice-based RT trials, except in the left PreCUN, where there is a marked increase in activity during fast trials, underscoring its potential role in the objective evaluation of confidence. In comparison, DP activity is generally higher for slow RT trials in various brain regions across participants, although these differences are not statistically significant. Solid (dashed) lines: averages across participants (best-fitted participant). Shaded areas: 95% confidence. Filled markers above horizontal axis: time points where significant differences were found between the averaged conditions ($p < 0.05$). Left PreCUN shows increased SS activity during fast RT trials, suggesting objective evaluation of decision confidence whereas no significant DP activity difference was observed.

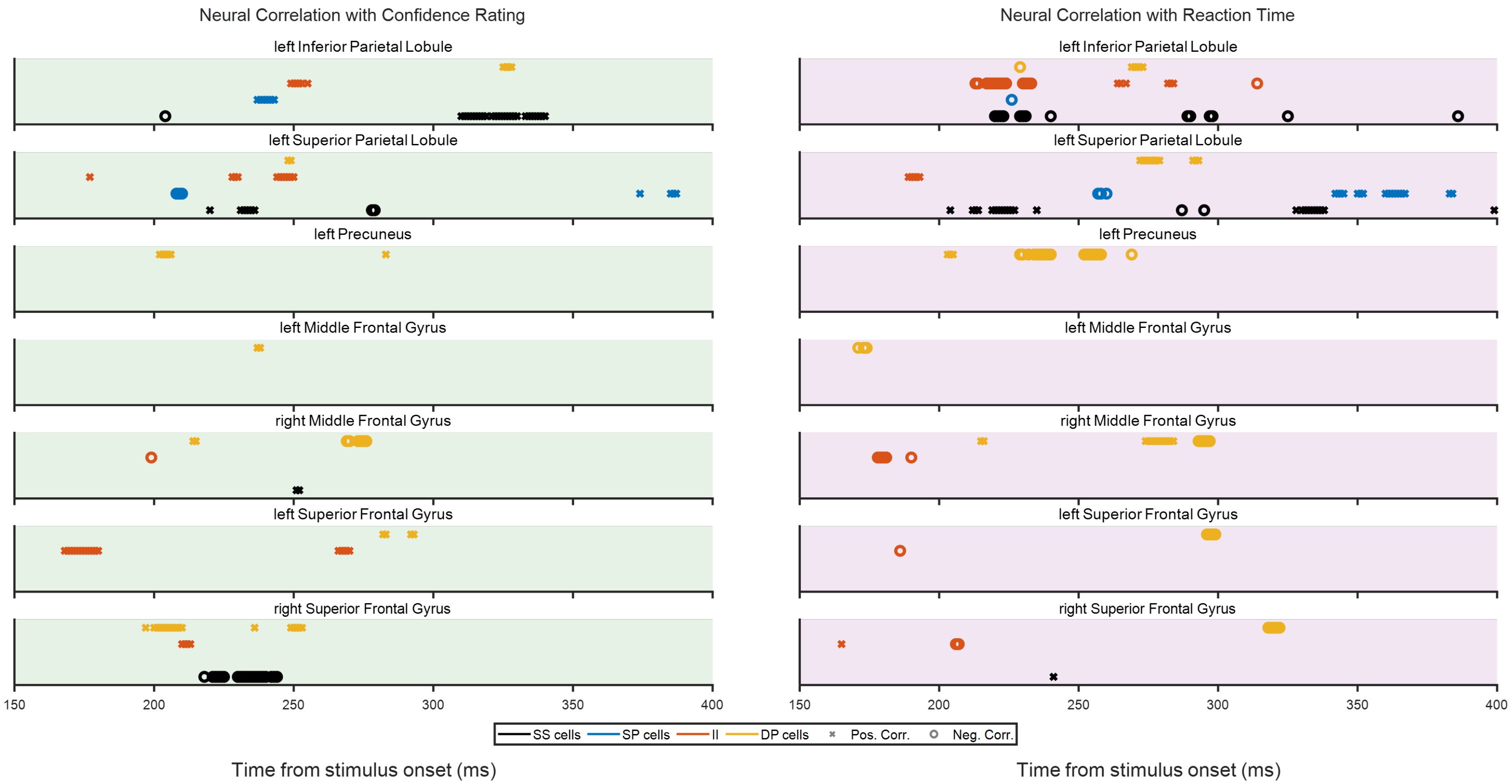

Fig. S16. Correlations suggest SS activity in left IPL relate to subjective decision confidence and right SFG to decision uncertainty, with left PreCUN linked to faster choice-based RTs (objective confidence proxy) and left SPL to slower choice-based RTs (objective uncertainty proxy). Correlation of estimated source activity (SS, SP, and DP and II neural populations) with subjective confidence ratings and choice-based RTs in high confidence trials, delineating the distinct roles of these regions in encoding cognitive aspects of confidence and decision-making speed. Trial-by-trial DCM analysis using support vector regression (SVR) reveals that SS activity in left IPL correlates with subjective confidence and those in the right SFG with uncertainty, while activities in the left PreCUN and the left SPL are associated with faster and slower RTs, respectively, hinting at their roles in objective confidence and uncertainty.

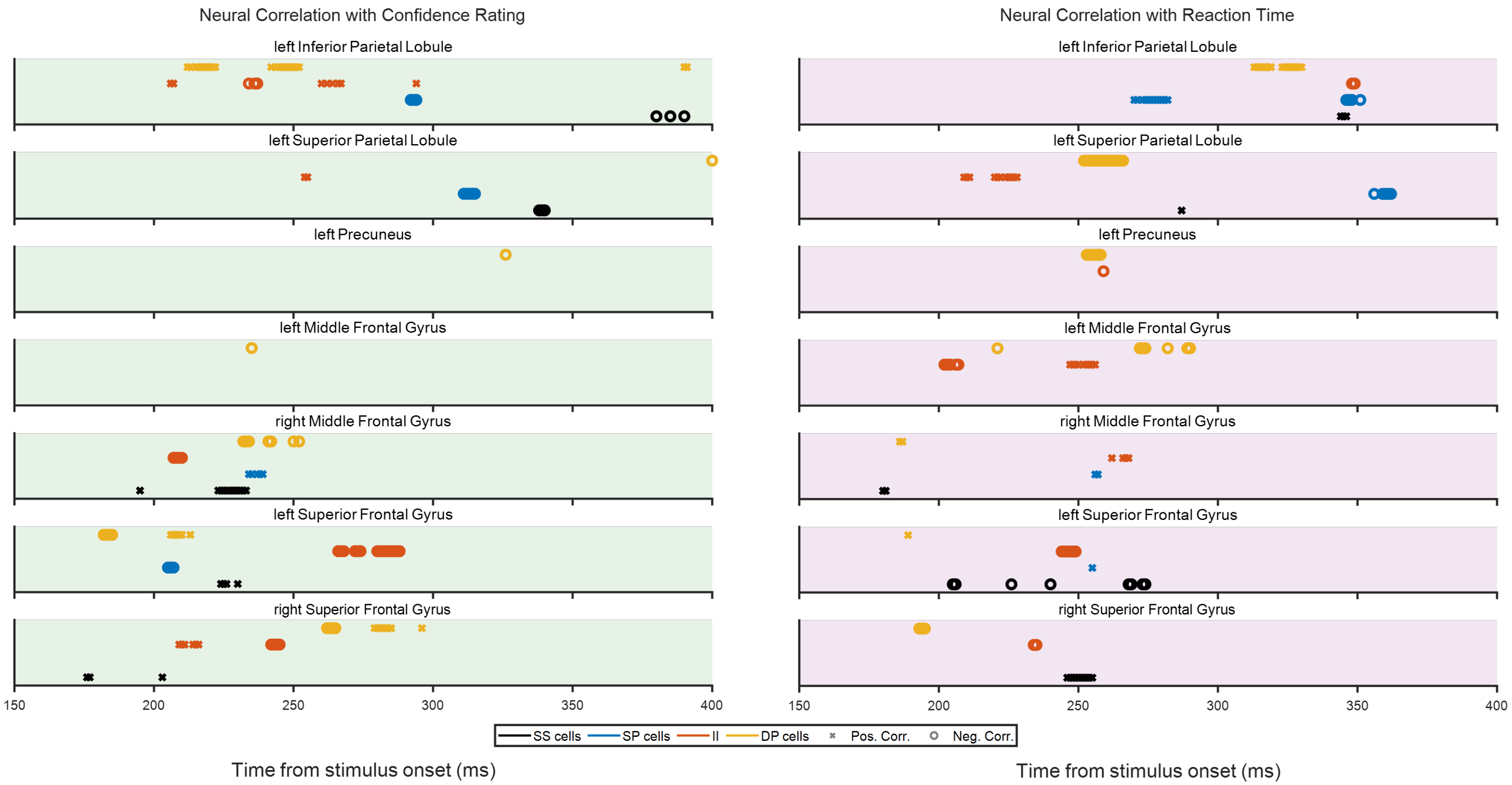

Fig. S17. Estimated activities of II in the left SFG are associated with the encoding of subjective decision uncertainty in low confidence rating trials indicated by trial-by-trial DCM. Correlation of estimated source activity (SS, SP, and DP and II neural populations) with subjective confidence ratings and choice-based RTs during low confidence rating trials, providing insights into the dynamic neural processes underpinning confidence and decision-making speed. Trial-by-trial DCM analysis using SVR reveals that II activity in left SFG correlates with subjective decision uncertainty.

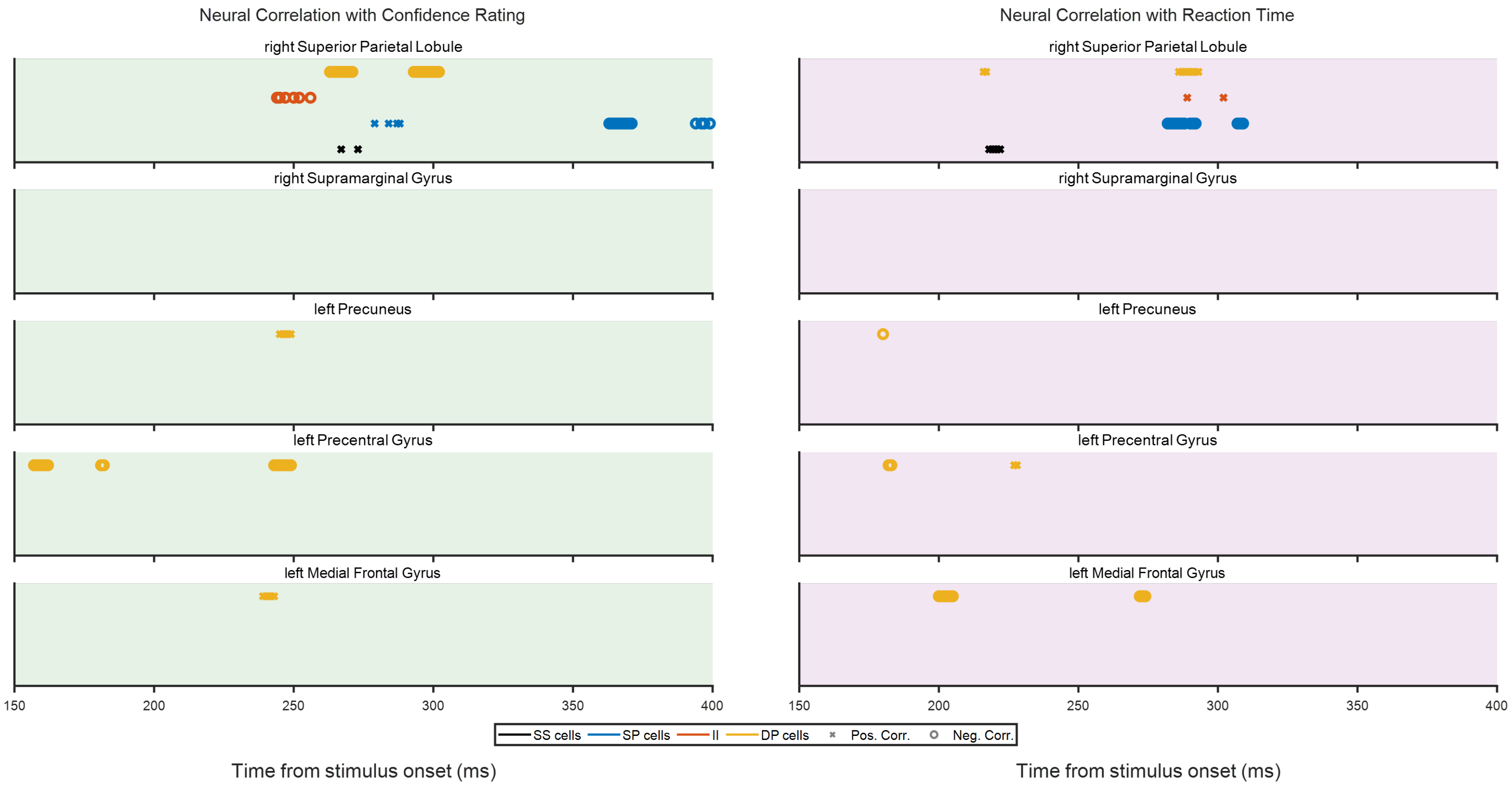

Fig. S18. Estimated activities of SP and DP neural populations in right SPL correlate with subjective decision uncertainty. Correlation of estimated source activity (SS, SP, and DP and II neural populations) with subjective confidence ratings and choice-based RTs, delineating the association neural activity within neural populations confidence levels and decision-making speed during trials with fast choice-based RTs. Trial-by-trial DCM analysis using SVR reveals that SP and DP neural population activity in right SPL correlates with subjective decision uncertainty.

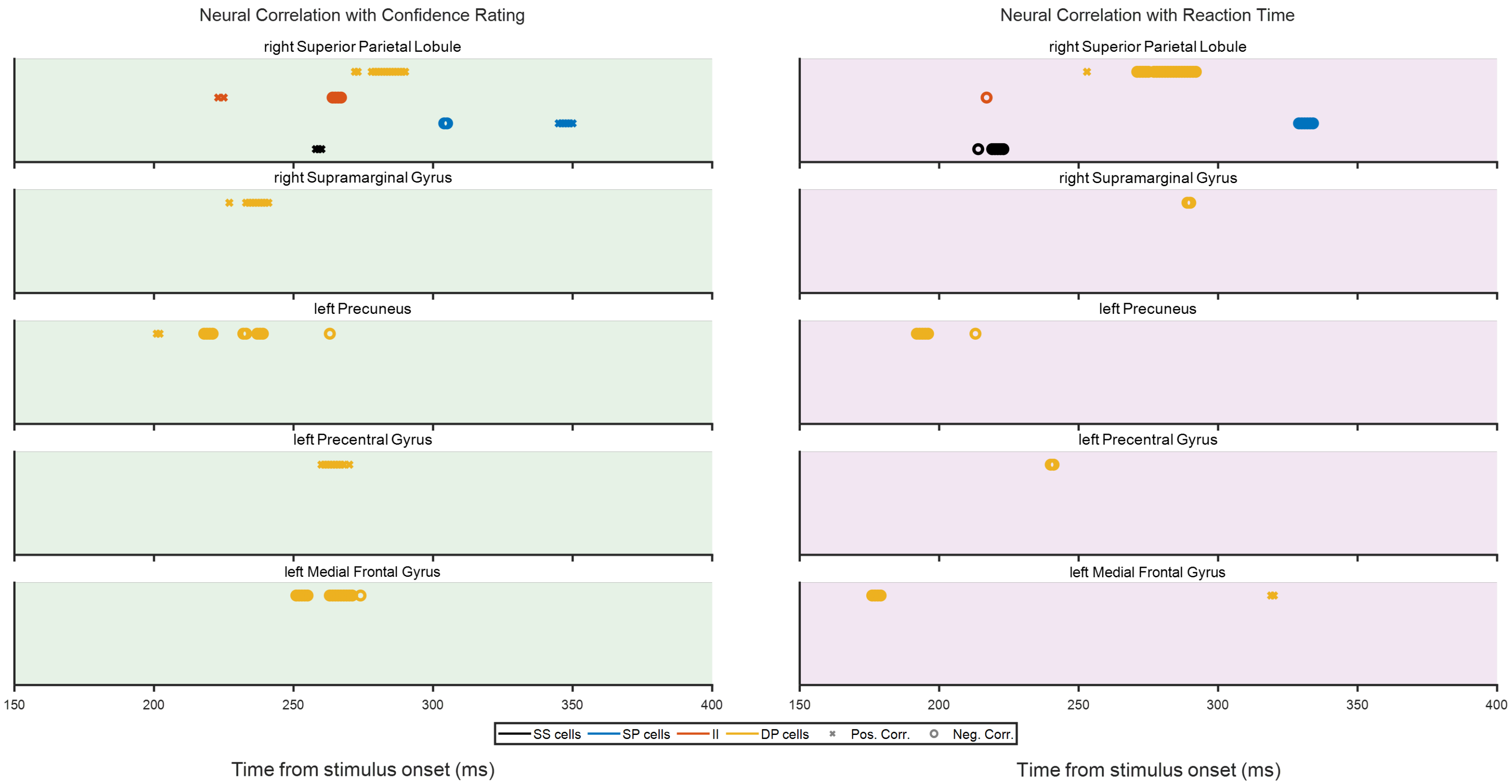

Fig. S19. In slow RT trials, estimated DP activity in left PreCUN correlates with confidence rating, while estimated activities of DP neural poulation in right SPL correlates with faster choice-based RTs. Correlation between estimated source activity and both subjective confidence ratings and choice-based RTs, during trials with slow choice-based RTs, highlighting the neural correlates of confidence and decision speed. Trial-by-trial DCM analysis using SVR shows that DP neural population activity in left PreCUN correlates with confidence rating suggesting its role in subjective decision confidence whereas DP activity in right SPL correlates with faster choice-based RTs hinting its role in objective decision confidence.

References

Bang, D., & Fleming, S. M. (2018). Distinct encoding of decision confidence in human medial prefrontal cortex. *Proceedings of the National Academy of Sciences*, *115*(23), 6082–6087. https://doi.org/10.1073/pnas.1800795115

Bastos, A. M., Usrey, W. M., Adams, R. A., Mangun, G. R., Fries, P., & Friston, K. J. (2012). Canonical Microcircuits for Predictive Coding. *Neuron*, *76*(4), 695–711. https://doi.org/10.1016/j.neuron.2012.10.038

Bonferroni, C. E. (1936). Teoria statistica delle classi e calcolo delle probabilita. *Pubblicazioni Del R Istituto Superiore Di Scienze Economiche e Commericiali Di Firenze*, *8*, 3–62.

Breiman, L. (2001). Random Forests. *Machine Learning*, *45*(1), 5–32. https://doi.org/10.1023/A:1010933404324

David, O., Kiebel, S. J., Harrison, L. M., Mattout, J., Kilner, J. M., & Friston, K. J. (2006). Dynamic causal modeling of evoked responses in EEG and MEG. *NeuroImage*, *30*(4), 1255–1272. https://doi.org/10.1016/j.neuroimage.2005.10.045

De Martino, F., Olman, C., & Valente, G. (2015). Information Decoding from fMRI Images. In K. Uludag, K. Ugurbil, & L. Berliner (Eds.), *fMRI: From Nuclear Spins to Brain Functions* (pp. 661–697). Springer US. https://doi.org/10.1007/978-1-4899-7591-1_23

Delorme, A., & Makeig, S. (2004). EEGLAB: An open source toolbox for analysis of single-trial EEG dynamics including independent component analysis. *Journal of Neuroscience Methods*, *134*(1), 9–21. https://doi.org/10.1016/j.jneumeth.2003.10.009

Donner, T. H., Siegel, M., Fries, P., & Engel, A. K. (2009). Buildup of Choice-Predictive Activity in Human Motor Cortex during Perceptual Decision Making. *Current Biology*, *19*(18), 1581–1585. https://doi.org/10.1016/j.cub.2009.07.066

Drucker, H., Burges, C. J. C., Kaufman, L., Smola, A., & Vapnik, V. (1996). Support Vector Regression Machines. *Advances in Neural Information Processing Systems*, *9*. https://papers.nips.cc/paper_files/paper/1996/hash/d38901788c533e8286cb6400b40b386d-Abstract.html

Efron, B. (1979). Bootstrap Methods: Another Look at the Jackknife. *The Annals of Statistics*, *7*(1), 1–26. https://doi.org/10.1214/aos/1176344552

Fisher, R. A. (1935). *The design of experiments* (pp. xi, 251). Oliver & Boyd.

FitzGerald, T. H. B., Moran, R. J., Friston, K. J., & Dolan, R. J. (2015). Precision and neuronal dynamics in the human posterior parietal cortex during evidence accumulation. *NeuroImage*, *107*, 219–228. https://doi.org/10.1016/j.neuroimage.2014.12.015

Fleming, S. M., van der Putten, E. J., & Daw, N. D. (2018). Neural mediators of changes of mind about perceptual decisions. *Nature Neuroscience*, *21*(4), Article 4. https://doi.org/10.1038/s41593-018-0104-6

Gherman, S., & Philiastides, M. G. (2015). Neural representations of confidence emerge from the process of decision formation during perceptual choices. *NeuroImage*, *106*, 134–143. https://doi.org/10.1016/j.neuroimage.2014.11.036

Gherman, S., & Philiastides, M. G. (2018). Human VMPFC encodes early signatures of confidence in perceptual decisions. *eLife*, *7*, e38293. https://doi.org/10.7554/eLife.38293

Gherman, S., & Philiastides, M. G. (2020). *Simultaneous EEG-fMRI - Confidence in perceptual decisions* [Dataset]. Openneuro. https://doi.org/10.18112/OPENNEURO.DS002739.V1.0.0

Hastie, T., Tibshirani, R., & Friedman, J. (2009). *The Elements of Statistical Learning*. Springer. https://doi.org/10.1007/978-0-387-84858-7

Hebart, M. N., Schriever, Y., Donner, T. H., & Haynes, J.-D. (2016). The Relationship between Perceptual Decision Variables and Confidence in the Human Brain. *Cerebral Cortex*, *26*(1), 118–130. https://doi.org/10.1093/cercor/bhu181

Heereman, J., Walter, H., & Heekeren, H. R. (2015). A task-independent neural representation of subjective certainty in visual perception. *Frontiers in Human Neuroscience*, *9*. https://www.frontiersin.org/articles/10.3389/fnhum.2015.00551

Hilgenstock, R., Weiss, T., & Witte, O. W. (2014). You'd Better Think Twice: Post-Decision Perceptual Confidence. *NeuroImage*, *99*, 323–331. https://doi.org/10.1016/j.neuroimage.2014.05.049

Hinch, E. J. (1991). *Perturbation Methods*. Cambridge University Press.

Hoven, M., Brunner, G., de Boer, N. S., Goudriaan, A. E., Denys, D., van Holst, R. J., Luigjes, J., & Lebreton, M. (2022). Motivational signals disrupt metacognitive signals in the human ventromedial prefrontal cortex. *Communications Biology*, *5*(1), Article 1. https://doi.org/10.1038/s42003-022-03197-z

IBM Corp. (2020). *IBM SPSS Statistics for Windows* (Version 27.0) [Computer software].

Jaeger, C., Glim, S., Dimulescu, C., Ries, A., Sorg, C., & Wohlschläger, A. (2020). Segregated Co-activation Patterns in the Emergence of Decision Confidence

During Visual Perception. *Frontiers in Systems Neuroscience*, *14*. https://www.frontiersin.org/articles/10.3389/fnsys.2020.557693

James, G., Witten, D., Hastie, T., & Tibshirani, R. (2013). *An Introduction to Statistical Learning* (Vol. 103). Springer. https://doi.org/10.1007/978-1-4614-7138-7

Kepecs, A., Uchida, N., Zariwala, H. A., & Mainen, Z. F. (2008). Neural correlates, computation and behavioural impact of decision confidence. *Nature*, *455*(7210), Article 7210. https://doi.org/10.1038/nature07200

Kiani, R., & Shadlen, M. N. (2009). Representation of Confidence Associated with a Decision by Neurons in the Parietal Cortex. *Science*, *324*(5928), 759–764. https://doi.org/10.1126/science.1169405

Lamme, V. A. F., & Roelfsema, P. R. (2000). The distinct modes of vision offered by feedforward and recurrent processing. *Trends in Neurosciences*, *23*(11), 571–579. https://doi.org/10.1016/S0166-2236(00)01657-X

Li, S., & Yang, F. (2012). Task-dependent uncertainty modulation of perceptual decisions in the human brain. *European Journal of Neuroscience*, *36*(12), 3732–3739. https://doi.org/10.1111/ejn.12006

Morales, J., Lau, H., & Fleming, S. M. (2018). Domain-General and Domain-Specific Patterns of Activity Supporting Metacognition in Human Prefrontal Cortex. *Journal of Neuroscience*, *38*(14), 3534–3546. https://doi.org/10.1523/JNEUROSCI.2360-17.2018

Penny, W. D., Friston, K. J., Ashburner, J. T., Kiebel, S. J., & Nichols, T. E. (2011). *Statistical Parametric Mapping: The Analysis of Functional Brain Images*. Elsevier.

Pereira, M., Faivre, N., Iturrate, I., Wirthlin, M., Serafini, L., Martin, S., Desvachez, A., Blanke, O., Van De Ville, D., & Millán, J. del R. (2020). Disentangling the origins of confidence in speeded perceptual judgments through multimodal imaging. *Proceedings of the National Academy of Sciences*, *117*(15), 8382–8390. https://doi.org/10.1073/pnas.1918335117

Pinotsis, D. A., Schwarzkopf, D. S., Litvak, V., Rees, G., Barnes, G., & Friston, K. J. (2013). Dynamic causal modelling of lateral interactions in the visual cortex. *NeuroImage*, *66*, 563–576. https://doi.org/10.1016/j.neuroimage.2012.10.078

Qiu, L., Su, J., Ni, Y., Bai, Y., Zhang, X., Li, X., & Wan, X. (2018). The neural system of metacognition accompanying decision-making in the prefrontal cortex. *PLOS Biology*, *16*(4), e2004037. https://doi.org/10.1371/journal.pbio.2004037

Shapiro, A. D., & Grafton, S. T. (2020). Subjective value then confidence in human ventromedial prefrontal cortex. *PLOS ONE*, *15*(2), e0225617. https://doi.org/10.1371/journal.pone.0225617

Trujillo-Barreto, N. J., Aubert-Vázquez, E., & Valdés-Sosa, P. A. (2004). Bayesian model averaging in EEG/MEG imaging. *NeuroImage*, *21*(4), 1300–1319. https://doi.org/10.1016/j.neuroimage.2003.11.008
